# A cooperative knock-on mechanism underpins Ca^2+^-selective cation permeation in TRPV channels

**DOI:** 10.1085/jgp.202213226

**Published:** 2023-03-21

**Authors:** Callum M. Ives, Neil J. Thomson, Ulrich Zachariae

**Affiliations:** 1 Computational Biology, School of Life Sciences, University of Dundee, Dundee, UK; 2 Biochemistry and Drug Discovery, School of Life Sciences, University of Dundee, Dundee, UK

## Abstract

The selective exchange of ions across cellular membranes is a vital biological process. Ca^2+^-mediated signaling is implicated in a broad array of physiological processes in cells, while elevated intracellular concentrations of Ca^2+^ are cytotoxic. Due to the significance of this cation, strict Ca^2+^ concentration gradients are maintained across the plasma and organelle membranes. Therefore, Ca^2+^ signaling relies on permeation through selective ion channels that control the flux of Ca^2+^ ions. A key family of Ca^2+^-permeable membrane channels is the polymodal signal-detecting transient receptor potential (TRP) ion channels. TRP channels are activated by a wide variety of cues including temperature, small molecules, transmembrane voltage, and mechanical stimuli. While most members of this family permeate a broad range of cations non-selectively, TRPV5 and TRPV6 are unique due to their strong Ca^2+^ selectivity. Here, we address the question of how some members of the TRPV subfamily show a high degree of Ca^2+^ selectivity while others conduct a wider spectrum of cations. We present results from all-atom molecular dynamics simulations of ion permeation through two Ca^2+^-selective and two non-selective TRPV channels. Using a new method to quantify permeation cooperativity based on mutual information, we show that Ca^2+^-selective TRPV channel permeation occurs by a three-binding site knock-on mechanism, whereas a two-binding site knock-on mechanism is observed in non-selective TRPV channels. Each of the ion binding sites involved displayed greater affinity for Ca^2+^ over Na^+^. As such, our results suggest that coupling to an extra binding site in the Ca^2+^-selective TRPV channels underpins their increased selectivity for Ca^2+^ over Na^+^ ions. Furthermore, analysis of all available TRPV channel structures shows that the selectivity filter entrance region is wider for the non-selective TRPV channels, slightly destabilizing ion binding at this site, which is likely to underlie mechanistic decoupling.

## Teaser

TRPV5 and TRPV6 are unique among TRP channels due to their high Ca^2+^ selectivity, while most other members of this ion channel family do not select for a specific cation type. Ives et al. used biomolecular simulations and in silico electrophysiology to determine the mechanism underlying this unusual Ca^2+^ selectivity.

## Introduction

The significance of Ca^2+^ in cellular function was first recognized by Sydney Ringer in 1883, who demonstrated that minute amounts of calcium were required for the contraction of cardiac muscle (Ringer, 1883). Ca^2+^ is now recognized as a versatile signaling agent, with cellular Ca^2+^ concentrations impacting a broad array of physiological processes ranging from cell proliferation to cell suicide (Carafoli, 2002; Berridge et al., 2003; Clapham, 2007; Patel, 2019). However, the cytoplasmic concentration of Ca^2+^ ions is usually kept low due to cytotoxic consequences (Bagur and Hajnóczky, 2017). Therefore, the controlled opening of channels in cellular and organellar membranes is one of the required mechanisms to allow the influx of this ion from the exoplasm and internal storage compartments into the cytoplasm; this subsequently initiates the Ca^2+^ signaling cascade. The question of how Ca^2+^ channels selectively permeate Ca^2+^ in low concentrations over vastly more abundant Na^+^ ions and yet conduct them at high rates has been a longstanding matter of fascination for ion channel researchers (Corry et al., 2001; Hille, 2001).

A key example of ion channels that mediate Ca^2+^ permeation across the cytoplasmic membrane is the transient receptor potential (TRP) channel superfamily. In their open state, these polymodal signal-detecting TRP channels allow the transmembrane flux of cations down their electrochemical gradient, thereby increasing the intracellular Ca^2+^ and Na^+^ concentration (Ramsey et al., 2006). The malfunction of TRP channels underlies a wide range of diseases, and they are therefore of immense biomedical importance, serving as drug targets for a variety of existing and candidate drugs (Moran, 2018).

TRP channels assemble primarily as homotetramers to form functional ion channels. A conserved structural feature across all TRP channels is the presence of six transmembrane helices (S1–S6) per subunit, forming two distinct transmembrane domains; a four-helix bundle comprising of helices S1–S4 forming the voltage-sensing like domain; and the pore-forming domain consisting of helices S5 and S6 (Hilton et al., 2019).

**Figure 1.**
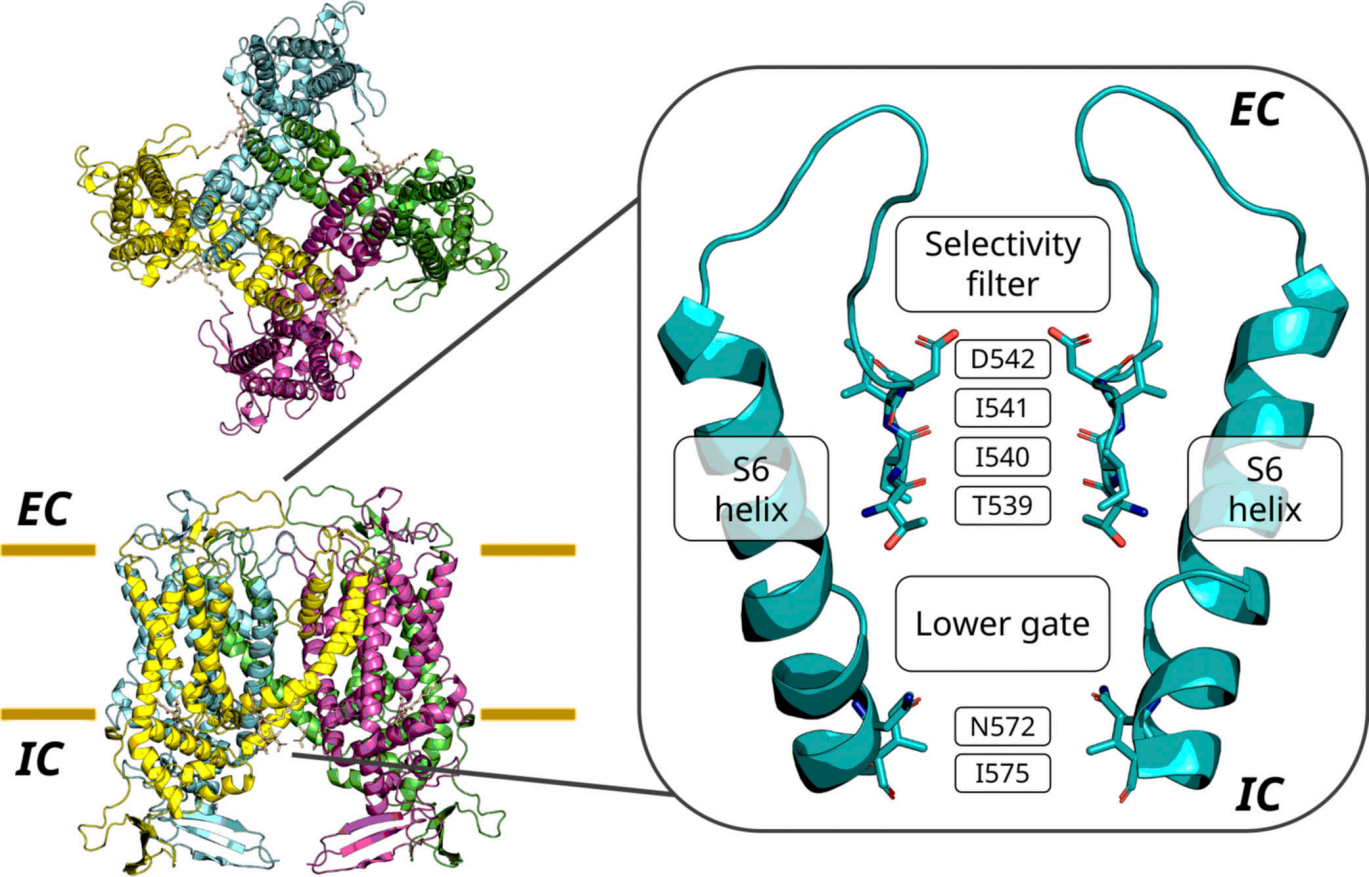
**Structure of the truncated construct of TRPV5 of *Oryctolagus cuniculus* used in this work, from the extracellular side (top left) and in the plane of the lipid bilayer (bottom left).** In this study, the pore is defined as the region between the constrictions of the channel, namely the top residue of the SF (referred to as the *α*-position of the SF) and the hydrophobic lower gate (right). EC, extracellular side; IC, intracellular side of the membrane.

A four-residue ion selectivity filter (SF) is located at the entrance of the channel pore (Fig. 1). In addition to this conserved transmembrane architecture, members of the TRP superfamily display highly diverse extramembrane loops and N- and C-terminal domains between the different subfamilies (Van Goor et al., 2020).

TRP channels are gated open by a particularly wide range of stimuli, which include temperature, small molecules, transmembrane voltage changes, and mechanical cues (Caterina et al., 1997; Tominaga et al., 1998; Caterina et al., 2000). This superfamily of genes can be divided into seven main subfamilies: TRPA (ankyrin), TRPC (canonical), TRPM (melastatin), TRPML (mucolipin), TRPN (no mechanoreceptor potential C), TRPP (polycystin), and TRPV (vanilloid). It should be noted, however, that several less well-characterized TRP subfamilies have also been reported, including the TRPY (Palmer et al., 2001; Zhou et al., 2003; Chang et al., 2010), TRPVL (Peng et al., 2015), and TRPS (Himmel et al., 2020) subfamilies. The TRPV channels are the most intensely studied channel subfamily.

While they are all cation selective, most TRP channels electrophysiologically characterized to date show only limited discrimination between cation types, as well as between divalent and monovalent cations. However, the TRPV5 and TRPV6 channels are unique due to their high selectivity for Ca^2+^ cations over Na^+^ cations (*P*_*Ca*_*/P*_*Na*_ ∼100:1 from reversal potential measurements; Vennekens et al., 2000; Yue et al., 2001). Phylogenetic analysis has demonstrated that the TRPV5 and TRPV6 channels of vertebrates originated from an ancestral TRPV5/6 gene, which then diverged to form TRPV5 and TRPV6 from a duplication event after speciation (Flores-Aldama et al., 2000). Both of these channels are constitutively active due to basal levels of phosphatidylinositol 4,5-bisphosphate (PI(4,5)P_2_) in the cellular membrane and play a key role in Ca^2+^ homeostasis in the body (Van Goor et al., 2017). Despite their characteristic Ca^2+^ selectivity, both channels have been shown to permeate monovalent cations such as Na^+^ when divalent cations are absent (Nilius et al., 2000; Vennekens et al., 2000; Voets et al., 2003; Bödding and Flockerzi, 2004). By contrast, the remaining members of the TRPV subfamily, TRPV1–4, permeate both Ca^2+^ and Na^+^ cations, even in the presence of high Ca^2+^ concentrations, although they are still slightly Ca^2+^ selective, with a permeability ratio *P*_*Ca*_*/P*_*Na*_ ∼10:1. These channels gate in response to a number of stimuli, including raised temperature—in particular the archetypal member TRPV1 (Caterina et al., 1997; Tominaga et al., 1998; Caterina et al., 2000), which has led to TRPV1–4 being referred to as thermoTRPV channels—as well as endogenous and exogenous ligands.

In recent years, MD simulations have been successfully employed to shed light on ion channel function and the mode of action of channel-acting drugs in atomistic detail, for instance on K^+^ channels (Köpfer et al., 2014; Kopec et al., 2018), Na^+^ channels (Ulmschneider et al., 2013; Ke et al., 2014), Cl^−^ channels (McKiernan et al., 2020; Chavan et al., 2020), and ligand-gated ion channels (Sauguet et al., 2013). However, in the case of Ca^2+^-permeating channels, the conventional point-charge models used to describe uncoordinated Ca^2+^ ions have historically been inaccurate due to the neglected effects of electronic polarization (Kohagen et al., 2014a). This has resulted in an over-estimation of the binding energies between Ca^2+^ and proteins, hindering an accurate study of Ca^2+^ permeation in channels. Previous efforts to resolve this overestimation have included polarizable force field approaches (Li et al., 2015) and rescaled Ca^2+^ charges (Kohagen et al., 2014a; Kohagen et al., 2014b). More recently, Zhang et al. (2020) published a new multisite Ca^2+^ model specifically optimized for Ca^2+^–protein interactions (Zhang et al., 2020). This model correctly replicated experimental values for the hydration-free energy and the number of coordinated water molecules in the first solvation shell and showed Ca^2+^–protein binding energies comparable to the quantum mechanical and polarizable models. The multisite Ca^2+^ model has previously been used to investigate ion permeation of Ca^2+^ in a range of channels, including the type-1 ryanodine receptor (Zhang et al., 2020; Liu et al., 2021), α-amino-3-hydroxy-5-methyl-4-isoxazolepropionic acid receptors (Schackert et al., 2022), and recently the E protein of SARS-CoV-2 (Antonides et al., 2022 *Preprint*).

In the present work, we set out to elucidate the molecular basis of Ca^2+^ selectivity and permeation in the TRPV channel subfamily. We conducted atomistic molecular dynamics (MD) simulations of TRPV channels under transmembrane voltage and compared the cation permeation mechanism observed in the Ca^2+^-selective TRPV5 and TRPV6 channels to the permeation mechanism in two exemplar non-selective TRPV channels, TRPV2 and TRPV3. In total, we observed 2,851 full ion traversals from 17.25 µs of MD simulations, allowing us to decipher the permeation mechanisms and principles of ion selectivity in the TRPV family with statistical power. Our findings suggest that ion conduction in TRPV channels proceeds via a cooperative knock-on mechanism involving multiple ion binding sites. The degree of cooperativity in ion permeation, linking the multiple binding sites, determines the degree of ion selectivity in the channels.

## Materials and methods

### TRPV system construction

Truncated TRPV simulation systems consisting of the membrane-domain of the channels were constructed as described in Table 1. The systems were built using the CHARMM-GUI server (Jo et al., 2008). The charged N- and C-terminal residues were neutralized by capping with acetyl (ACE) and *N*-methylamide (CT3) groups, respectively, and all missing non-terminal residues were modeled (Jo et al., 2014). In the case of the TRPV5 system, the parameters for PI(4,5) P_2_ were generated using the CHARMM General Force Field (Vanommeslaeghe, 2010) through the ligand reader and modeler in CHARMM-GUI (Kim et al., 2017).

**Table 1.** Summary of protein constructs used in this study

	PDB ID	Ortholog	Residue range	Upper-gate residue	Lower-gate residue	Reference
TRPV2	6BO4	*Rattus norvegicus*	327–691	E609	I642	Dosey et al., 2019
TRPV3	6PVP	*Mus musculus*	375–745	D641	I674	Singh et al., 2019
TRPV5	6DMU	*Oryctolagus cuniculus*	262–639 (and PI(4,5)P_2_)	D542	I575	Hughes et al., 2018
TRPV6	6BO8	*Homo sapiens*	262–638	D542	I575	McGoldrick et al., 2018

The structures were aligned in the membrane using the PPM server (Lomize et al., 2012), inserted into a 1-palmitoyl-2-oleoyl-sn-glycerol-3-phosphocholine bilayer of 150 × 150 Å size using the CHARMM-GUI membrane builder (Jo et al., 2007; Wu et al., 2014), and then solvated. Ions were added using GROMACS 2020.2 (Abraham et al, 2015; Lindahl et al, 2020) to neutralize any system charges and add ions to a concentration of either 150 mM NaCl, 150 mM CaCl_2_, or a mixture of 75 mM NaCl and 75 mM CaCl_2_. In the case of simulations containing Ca^2+^, the standard CHARMM36m Ca^2+^ ions were then replaced with the multisite Ca^2+^ model of Zhang et al. (2020). Hydrogen mass repartitioning of the system was used to allow the use of 4 fs time steps in simulations of NaCl solutions. The multisite Ca^2+^ model used for simulations of CaCl_2_, however, is incompatible with a 4 fs time step, and therefore any simulations including Ca^2+^ cations were performed with hydrogen mass repartitioning but at a time step of 2 fs. The protein was restrained in the open-state by applying harmonic restraints on the *α*-carbon atoms of the lower gate residues (see Table 1).

### MD simulations details

All simulations were performed using GROMACS 2020.2 (Abraham et al, 2015; Lindahl et al, 2020) and the CHARMM36m force field for the proteins, lipids, and ions (Huang et al., 2017). The TIP3P water model was used to model solvent molecules (Jorgensen et al., 1983). The system was minimized and equilibrated using the suggested equilibration inputs from CHARMM-GUI (Lee et al., 2016). In brief, the system was equilibrated using the isothermal-isobaric (NPT) ensemble for a total time of 1.85 ns with the force constraints on the system components being gradually released over six equilibration steps. The systems were then further equilibrated by performing a 15 ns simulation with no electric field applied. To prevent closing of the lower gate of the pore, harmonic restraints were applied to maintain the distance between the *α*-carbon atoms of the lower gate residues of each respective chain (Table 1). To drive ion permeation, an external electric field was applied by using the method of Aksimentiev and Schulten (2005) to production simulations with an E_0_ of −0.03 V nm^−1^; this resulted in a transmembrane voltage of ∼410 mV with negative polarity in the intracellular region. The temperature was maintained at 310 K using the Nosé-Hoover thermostat (Evans and Holian, 1985) and the pressure was maintained semi-isotropically at 1 bar using the Parrinello-Rahman barostat (Parrinello and Rahman, 1981). Periodic boundary conditions were used throughout the simulations. Long-range electrostatic interactions were modeled using the particle-mesh Ewald method (Darden et al., 1993) with a cutoff of 12 Å. The linear constraint solver algorithm (Hess et al., 1997) was used to constrain bonds with hydrogen atoms. All individual simulations were 250 ns long and repeated five times for each system, as summarized in Table 2. Additional details for all simulations of Ca^2+^-selective and non-selective TRPV channels are described in Tables S1 and S2, respectively, and details for additional control simulations are described in Table S3.

**Table 2.** Summary of simulation details of Ca^2+^-selective and non-selective TRPV channels

Protein	Ion solution	Voltage	Simulation duration
TRPV5	150 mM CaCl_2_	−410 mV	5 × 250 ns
	150 mM NaCl	−410 mV	5 × 250 ns
	75 mM CaCl_2_ + 75 mM NaCl	−410 mV	5 × 250 ns
TRPV6	150 mM CaCl_2_	−410 mV	5 × 250 ns
	150 mM NaCl	−410 mV	5 × 250 ns
	75 mM CaCl_2_ + 75 mM NaCl	−410 mV	5 × 250 ns
TRPV2	150 mM CaCl_2_	−410 mV	5 × 250 ns
	150 mM NaCl	−410 mV	5 × 250 ns
	75 mM CaCl_2_ + 75 mM NaCl	−410 mV	5 × 250 ns
TRPV3	150 mM CaCl_2_	−410 mV	5 × 250 ns
	150 mM NaCl	−410 mV	5 × 250 ns
	75 mM CaCl_2_ + 75 mM NaCl	−410 mV	5 × 250 ns

### Simulation analysis

Analysis of MD trajectory data was performed using in-house written Python scripts, utilizing GROMACS modules (Abraham et al, 2015; Lindahl et al, 2020), the SciPy library of tools (Oliphant, 2007; Pérez and Granger, 2007; Millman and Aivazis, 2011; Van der Walt et al., 2011), and MDAnalysis (Michaud-Agrawal et al., 2011; Gowers et al., 2016). Analysis of the pore architecture was performed using the Channel Annotation Package (CHAP; Klesse et al, 2019). All plots were generated in Python using Matplotlib (Hunter, 2007) and Seaborn (Waskom et al., 2018).

### Calculating conductance and selectivity from in silico electrophysiology experiments

The conductance of the channels (*G*_*channel*_) was calculated according to Eq. 1, where *N*_*p*_ is the number of permeation events, *Q*_*ion*_ is the charge of the permeating ion in Coulomb, *t*_*traj*_ is the length of the trajectory, and *V*_*m*_ is the transmembrane voltage. The mean conductance and standard error were calculated from overlapping 50 ns windows of the trajectory.

$$G_{channel}=\frac{N_{p}\times Q_{ion}}{t_{traj}\times V_{m}}\text{.}$$ (1)

The selectivity (*P*_*Ca*_*/P*_*Na*_) was calculated as the ratio between the total sum of Ca^2+^ permeation events and the total sum of Na^+^ permeation events from fivefold replicated 250 ns simulations in dicationic solutions with 75 mM CaCl_2_ and 75 mM NaCl.

### Identification of cation binding sites from MD simulations of TRPV channels

Cation binding sites were identified by plotting timeseries of each permeating ion with respect to their position along the pore axis. To further validate this, the *Featurizer* function of PENSA (Vögele et al., 2021; Vögele et al., 2022) was used to identify the 12 most occupied ion binding sites, as determined by 3-D density maxima within 7 Å of the protein. This analysis was performed on a trajectory of concatenated fivefold replicated 250 ns simulations with a 200 ps time step from monocationic simulations.

### Calculating ion occupancy probabilities and residence times in the identified cation binding sites

The ion occupancy (*O*_*ion*_) of the identified cation binding sites was calculated by dividing the number of frames (*N*_*occupied*_), in which an ion’s center of geometry is within 3.5 Å of the center of geometry of the ion coordinating binding site atoms by the number of frames in the time window (*N*_*frames*_). These atoms for the respective binding sites were: the carboxylate oxygen atoms of the *α*-position residue of the SF for binding site A, the carbonyl oxygen atoms of the *β*- and *γ*-position residues of the SF for binding site B, and the terminal carbon atoms of the hydrocarbon side chain of the isoleucine of the hydrophobic lower gate (I575 in TRPV5) and the amide oxygen atoms of neighboring asparagine residue (N572 in TRPV5). A cut-off distance of 3.5 Å was chosen based on the maximum reported distance for calcium–oxygen interactions (Nayal and Di Cera, 1994; Dudev and Lim, 2003; Deng et al., 2006). The mean ion occupancies and standard error were calculated from non-overlapping 50 ns windows of the fivefold replicated 250 ns simulation trajectories with a 20 ps time step.

The ion residence times (*t*_*r*_) were calculated by averaging the amount of time an individual ion was located within 3.5 Å of the center of geometry of the ion coordinating binding site atoms. The mean *t*_*r*_ and standard error were calculated from fivefold replicated 250 ns simulation trajectories with a 20 ps time step.

### Characterizing permeation cooperativity through mutual information using state-specific information (SSI) from PENSA

To characterize the level of cooperativity in the knock-on permeation mechanisms in TRPV channels, we used PENSA to calculate the SSI shared between discrete state transitions in the occupancy distributions of each binding site (Thomson et al., 2021 *Preprint*; Vögele et al., 2021; Vögele et al., 2022). A timeseries distribution with a time step of 20 ps for each binding site was obtained, whereby for each frame, if an ion occupied the binding site, then this ion’s atom ID number was recorded, whereas if the binding site was unoccupied, an ID of −1 was recorded. The ID numbers were discrete, and changes between ID numbers in each binding site, therefore, represent discrete state transitions. By quantifying the mutual information shared between changes to the ID numbers in each site, we were able to determine whether ion transitions at one site were coupled to transitions at another during a 20 ps time interval. From this, we concluded whether cations are “knocking” each other, or dissociation occurred independently from one another. The time interval was iteratively optimized to keep noise and finite sampling effects to a minimum (see below). We found that both were smallest when we used an interval of 20 ps.

Similar to McClendon et al. (2009), we observed that finite sampling resulted in independent distributions sharing mutual information (McClendon et al., 2009; Pethel and Hahs, 2014). To overcome this, we calculated a statistical threshold for each simulation via randomly permuted copies of the original data. Random permutations of the original data maintained marginal probabilities for binding site occupation in each simulation while at the same time quantifying the effect of finite sampling on the measurement of SSI. SSI was then calculated between two independently permuted versions of the occupancy distribution for the minimum entropy binding site. Since the upper bound of mutual information between two variables is equal to the lowest entropy of those variables, we used the binding site corresponding to the lowest entropy for obtaining the threshold. This ensured that the portion of SSI which could be attributed to random noise between any two binding sites was always less than or equal to the SSI. This measurement was repeated 1,000 times to resolve a Gaussian distribution from which we obtained the 99% confidence threshold. We subtracted this threshold from the measured values to resolve excess mutual information, or excess SSI (*exSSI*), shared in discrete state transitions. As it is not possible to transfer negative information, negative *exSSI* values were corrected to a value of 0.

We also derived a maximum SSI value representing a theoretical upper limit for the information that can be shared between two binding sites, where *exSSI*_*max*_ is given by subtracting the random threshold from the minimum entropy of the two binding sites in question.

$$exSSI(A,B{)}_{\max}=\min\left(H\left(A\right),H\left(B\right)\right)-threshold\left(A,B\right)\text{.}$$ (2)

To quantify the interdependence of all three ion binding sites within the TRPV pores, the total correlation (TotCorr) was obtained using Eq. 3, where *H*(*A*), *H*(*B*), and *H*(*C*) represent the entropy of binding sites *A*, *B*, and *C*, respectively, and *H*(*A*, *B*, *C*) the joint entropy of binding sites *A*, *B*, and *C*.

TotCorr=H(A)+H(B)+H(C)−H(A,B,C). (3)

### Characterizing the architecture of the SF of TRPV channels

To determine the area formed between residues in the SF, the area of the quadrilateral between the adjacent chains was calculated on the X and Y axes. For this, we used the carboxylate oxygen atoms of the adjacent chains for the *α*-position residue, and the carbonyl oxygen atoms for the *β*-, *γ*-, and *δ*-position residues. To quantify the SF areas from our MD simulations, the mean and standard error were calculated from non-overlapping 50 ns windows of the fivefold replicated 250 ns trajectories with a 200 ps time step. Furthermore, the SF areas of all TRPV structures deposited in the Protein Data Bank (PDB), available as of February 4, 2022, were determined. A total of 101 structures were analyzed, with non-tetrameric structures or structures without all the atoms of interest modeled not included. The mean and SEM were calculated for all the available structures for any particular TRPV channel.

### Online supplemental material

**Figure S1.**
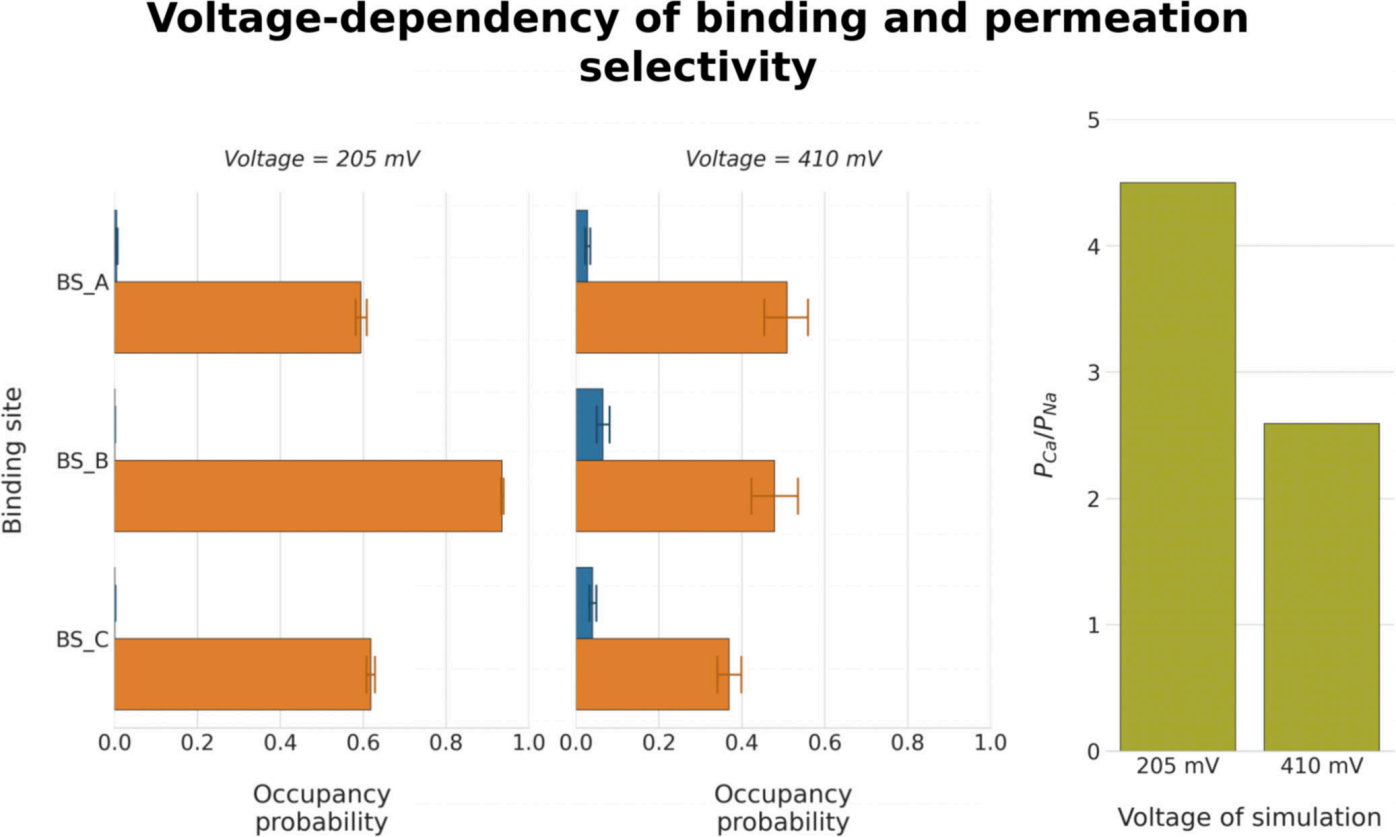
**The effect of high voltage on Ca**^**2+**^**-selectivity in simulations of TRPV5 in dicationic solutions.** Simulations performed at a lower voltage of ∼205 mV (left) resulted in an increased occupancy probability of Ca^2+^ cations (orange) and a reduced occupancy probability of Na^+^ cations (blue) compared to simulations at a higher voltage of ∼410 mV (center). This resulted in increased Ca^2+^ selectivity in lower voltage simulations, as summarized in the *P*_*Ca*_*/P*_*Na*_ value (right).

**Figure S2.**
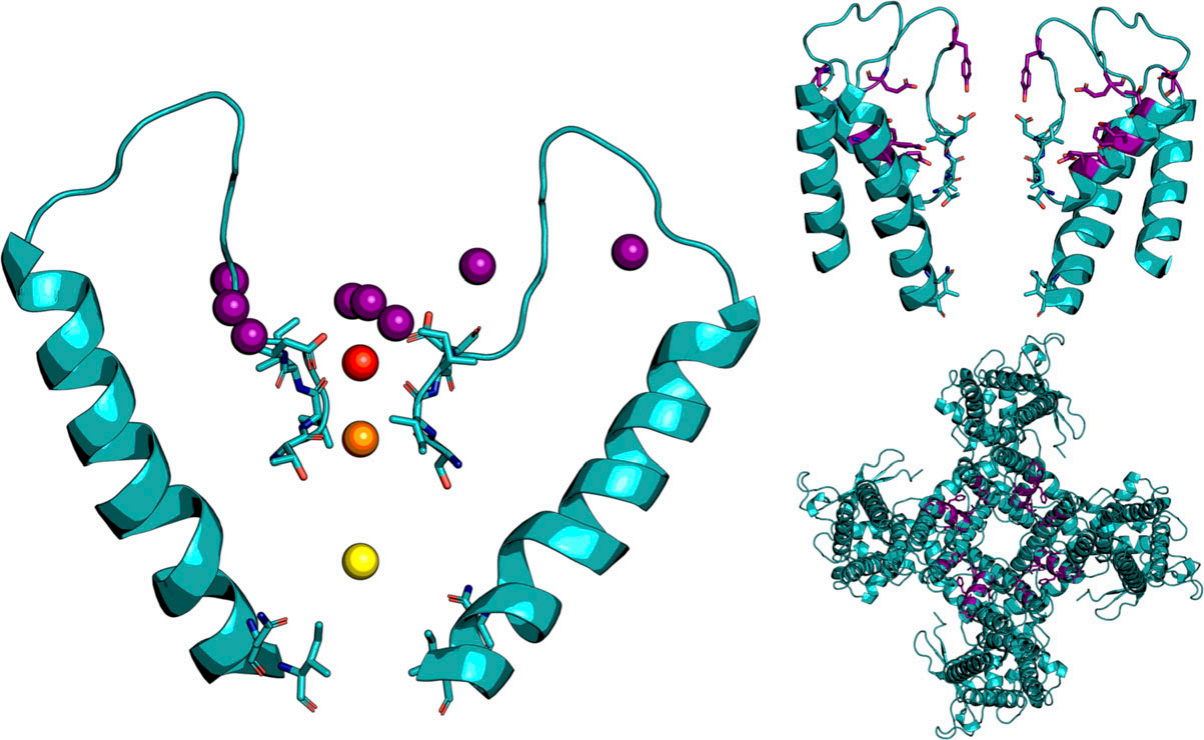
**Cation binding sites in TRPV5 identified by PENSA.** 3-D density maxima of Ca^2+^ cations within 7 Å of the protein was analyzed to identify 12 cation binding sites shown as pseudo-atoms (left). This analysis identified binding sites A (red), B (orange), and C (yellow) and several other “recruitment sites” (purple). The location of these recruitment sites (purple) help to attract cations and funnel them toward the pore entrance (top right and top left).

**Figure S3.**
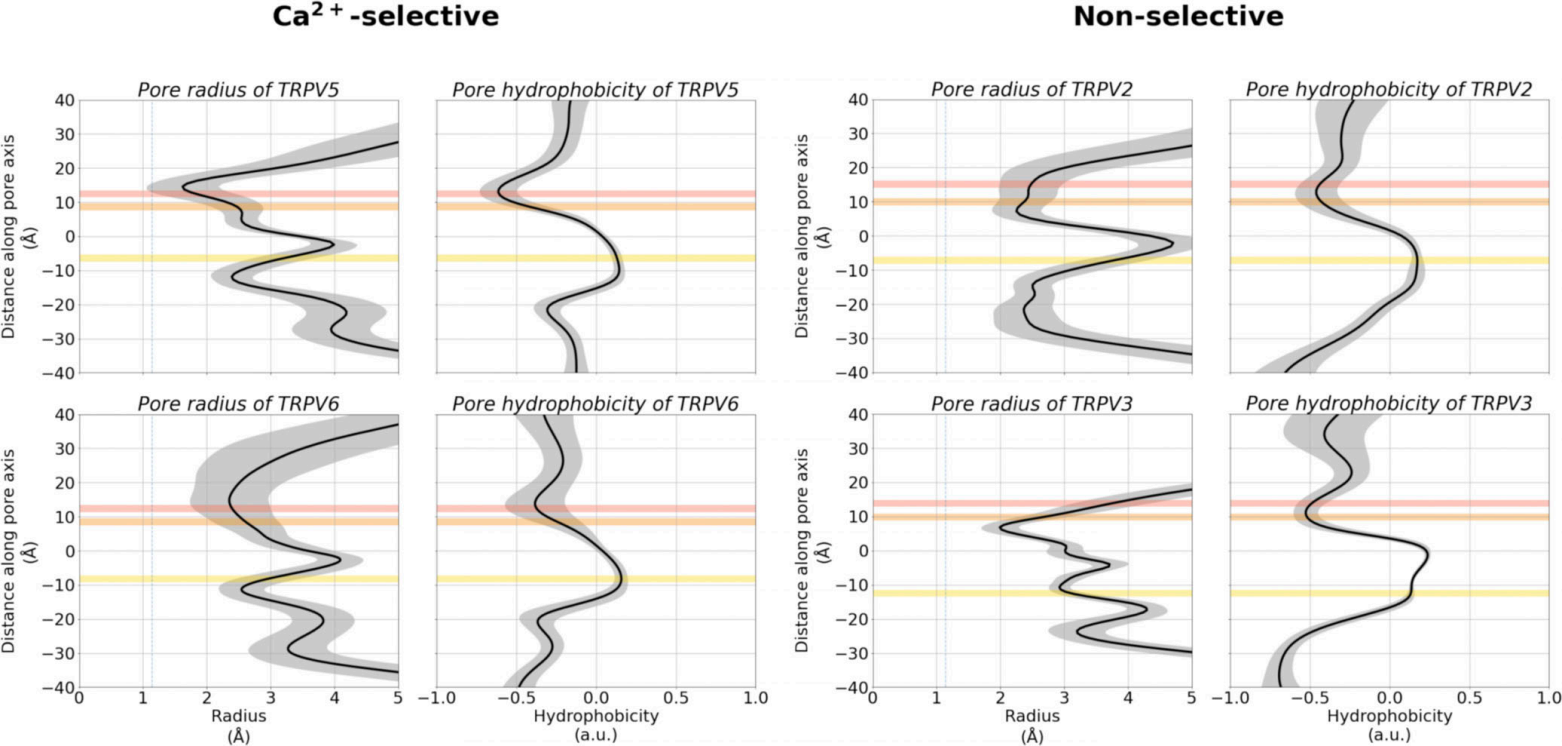
**Pore architecture of TRPV channels from MD simulations, showing the average radius and hydrophobicity of the channel withrespect to the relative *z* coordinate, obtained using CHAP (**Klesse et al, 2019**).** The mean radius or hydrophobicity (black) and SD (gray) were calculated from concatenated trajectories of fivefold replicated 250 ns simulations in 150 mM CaCl_2_ with a 200 ps time step. The shaded gray region represents the SD. The average position of binding sites A, B, and C are shown as shaded red, orange, and yellow regions, respectively. The dashed line in the pore radius plots indicates the radius of a dehydrated Ca^2+^ ion.

**Figure S4.**
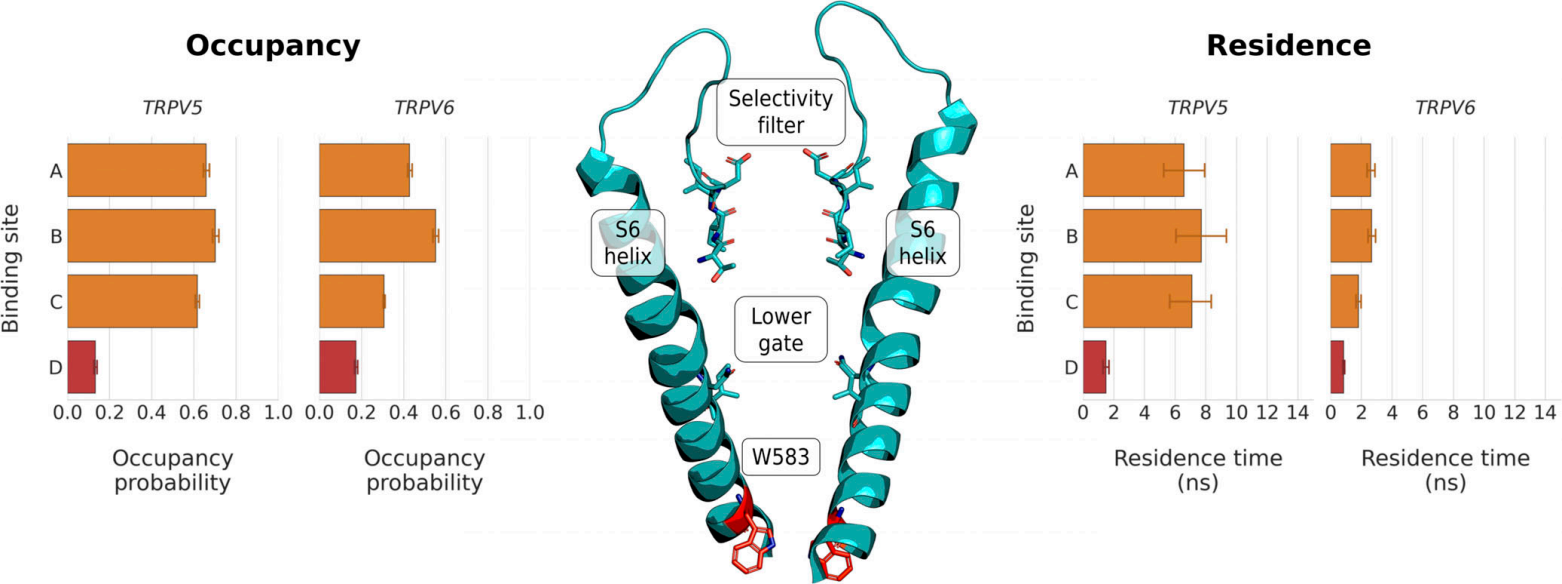
**W583 does not form a functionally important cation binding site in simulations of TRPV5 in 150 mM CaCl**_**2**_**.** W583 is located on the S6 helix, below the hydrophobic lower gate (center). Analysis of the occupancy probability (left) and the residence time (right) showed that the constriction formed by W583 does not coordinate Ca^2+^ cations as efficiently as binding sites A, B, and C in our simulations.

**Figure S5.**
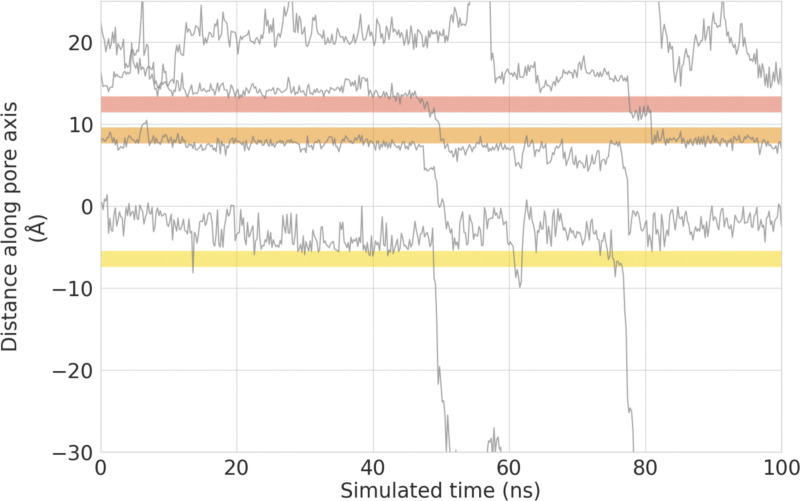
**Permeation traces of the *z*-coordinate of permeating Ca**^**2+**^ **cations over time in a lower concentration monocationic solution of 25 mM CaCl**_**2**_**.**

**Figure S6.**
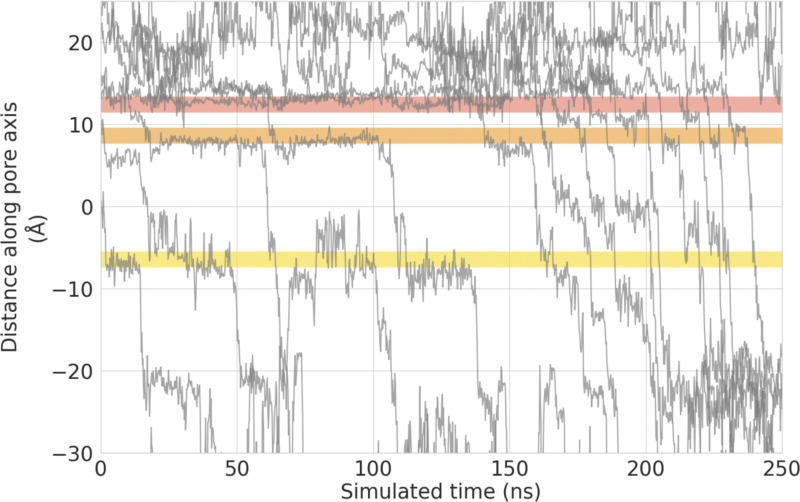
**Permeation traces of the *z*-coordinate of permeating Ca**^**2+**^ **cations over time in a dicationic solution of 75 mM CaCl**_**2**_ **and 75 mM NaCl at a lower voltage of −205 mV.**

**Figure S7.**
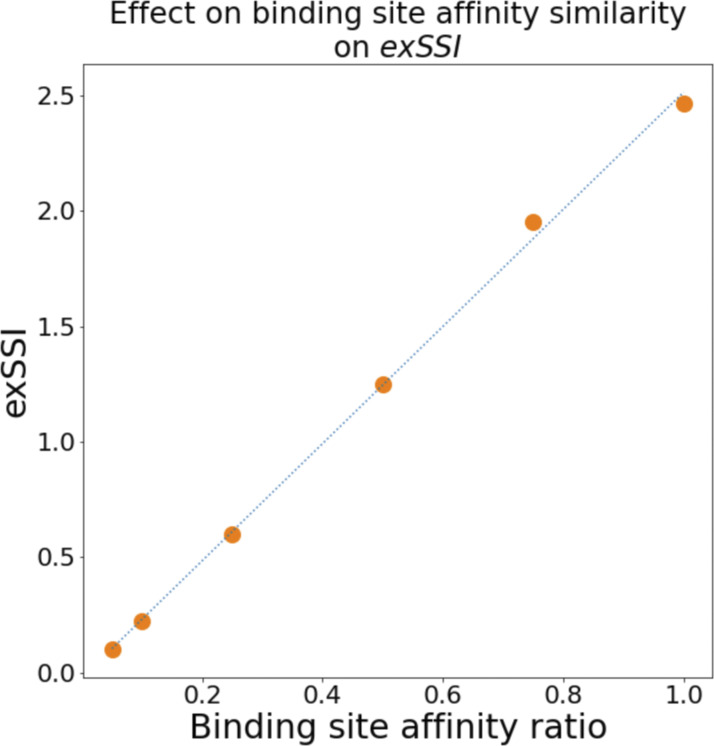
**The effect of similar binding site affinities on *exSSI* generated from a model of two consecutive binding sites.** When the binding site affinity of binding site *ε* was reduced relative to binding site *ζ*, the *exSSI* also decreased in a linear fashion.

**Figure S8.**
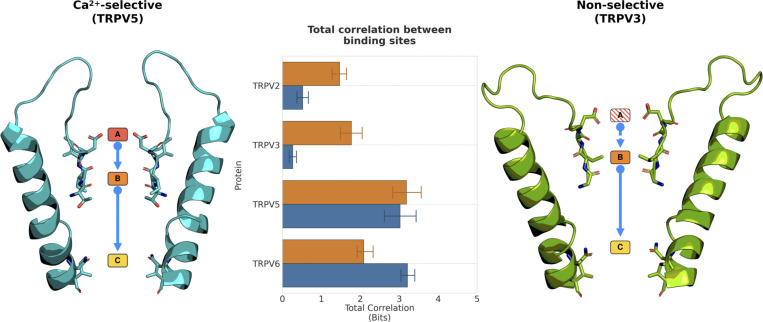
**Total correlation of cation permeation between cation binding sites from simulations of TRPV channels.** Comparison of the total correlation showed that the Ca^2+^-selective TRPV channels have a greater total correlation than non-selective TRPV channels (center; Ca^2+^, orange bars; Na^+^, blue bars). This greater total correlation in Ca^2+^-selective TRPV channels is a consequence of a knock-on mechanism between three binding sites (left). However, in non-selective TRPV channels, cation coordination at binding site A is reduced, resulting in reduced cooperativity, and a two-binding site knock-on mechanism between binding sites B and C only (right).

**Figure S9.**
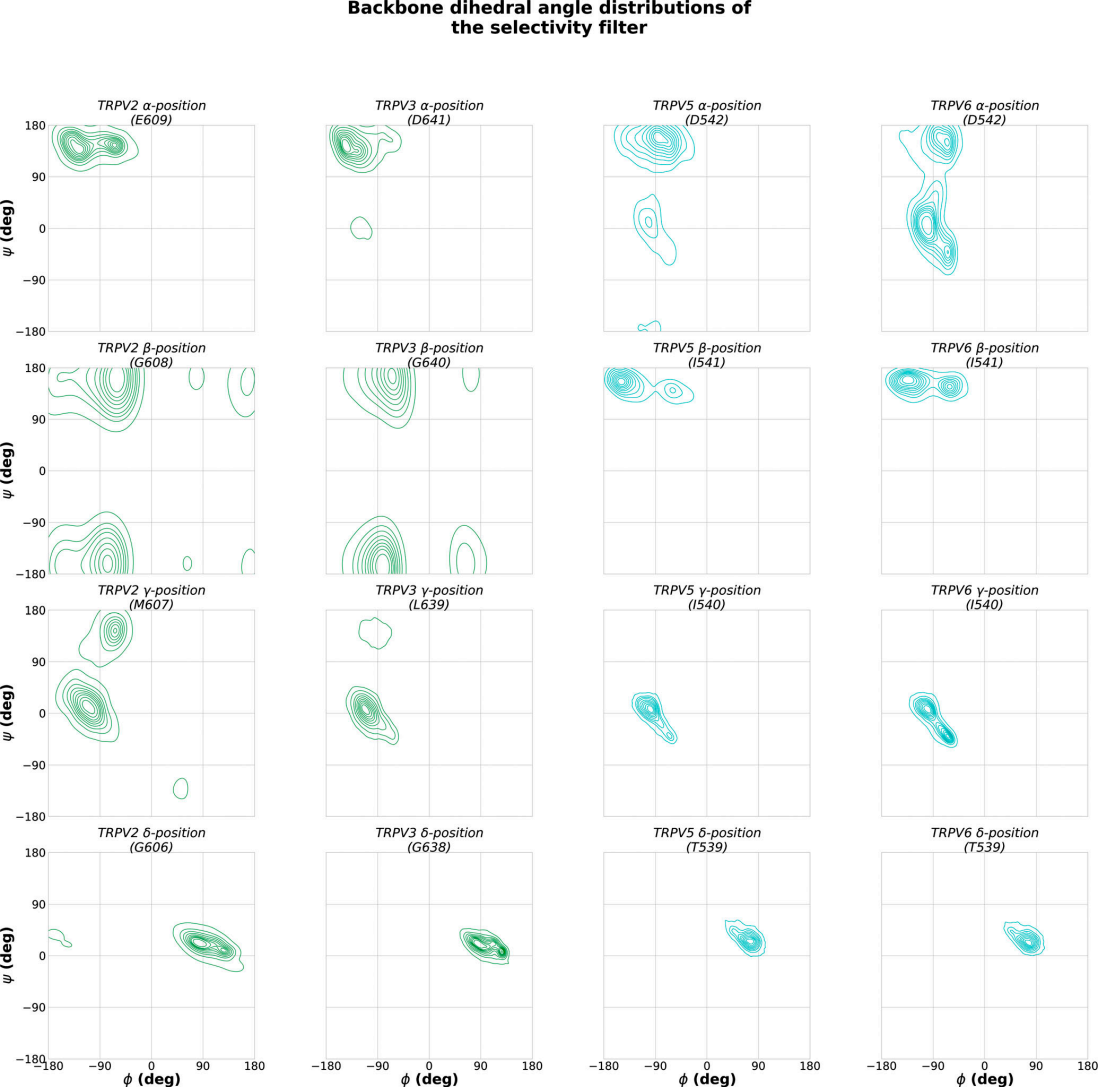
**Backbone dihedral angle distribution of SF residues in TRPV channels.** The *φ* and *ψ* angles were calculated across fivefold replicated 250 ns simulations of each TRPV channel in 150 mM CaCl_2_.

**Figure S10.**
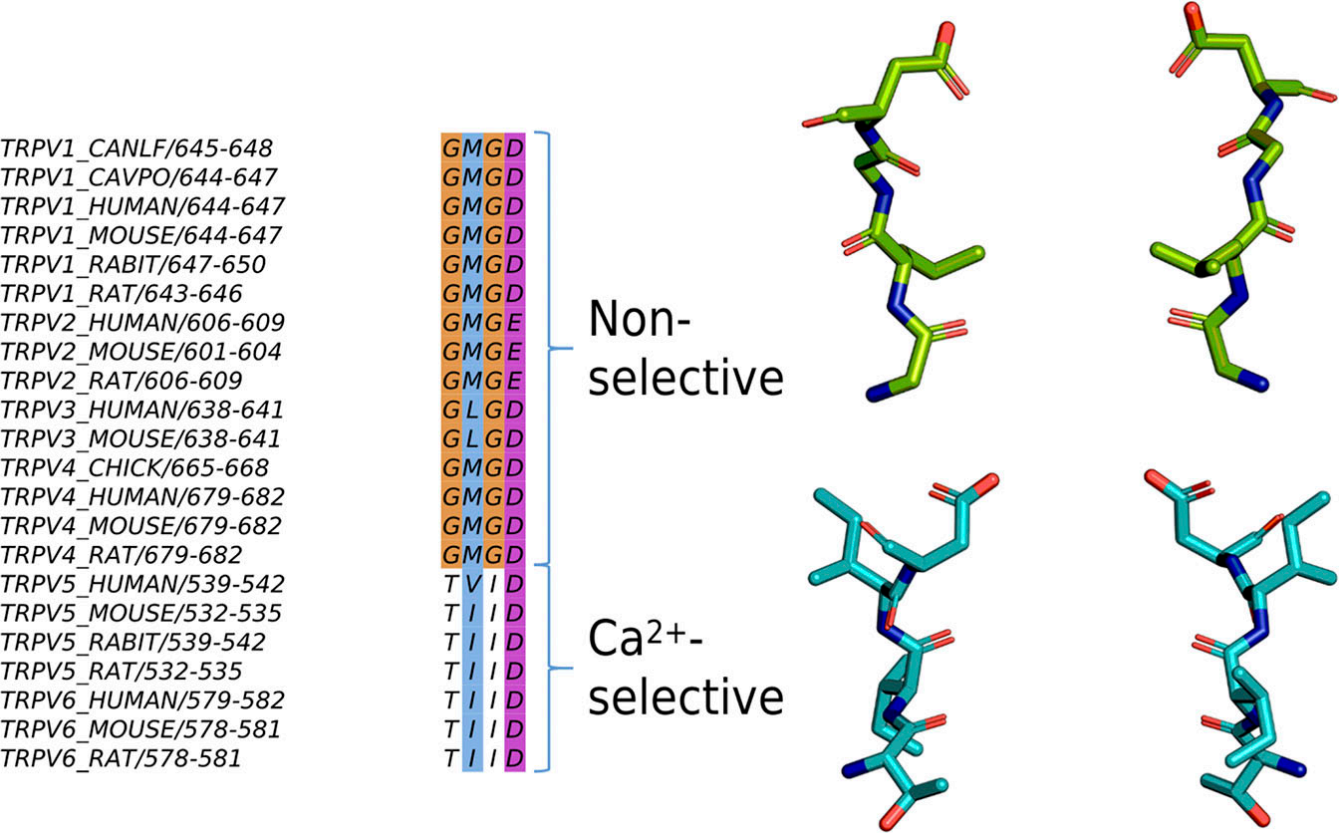
**Multiple sequence alignment of the SF domains of TRPV sequences deposited in the Swiss-Prot database.** Residues are colored according to the ClustalX coloring scheme.

**Figure S11.**
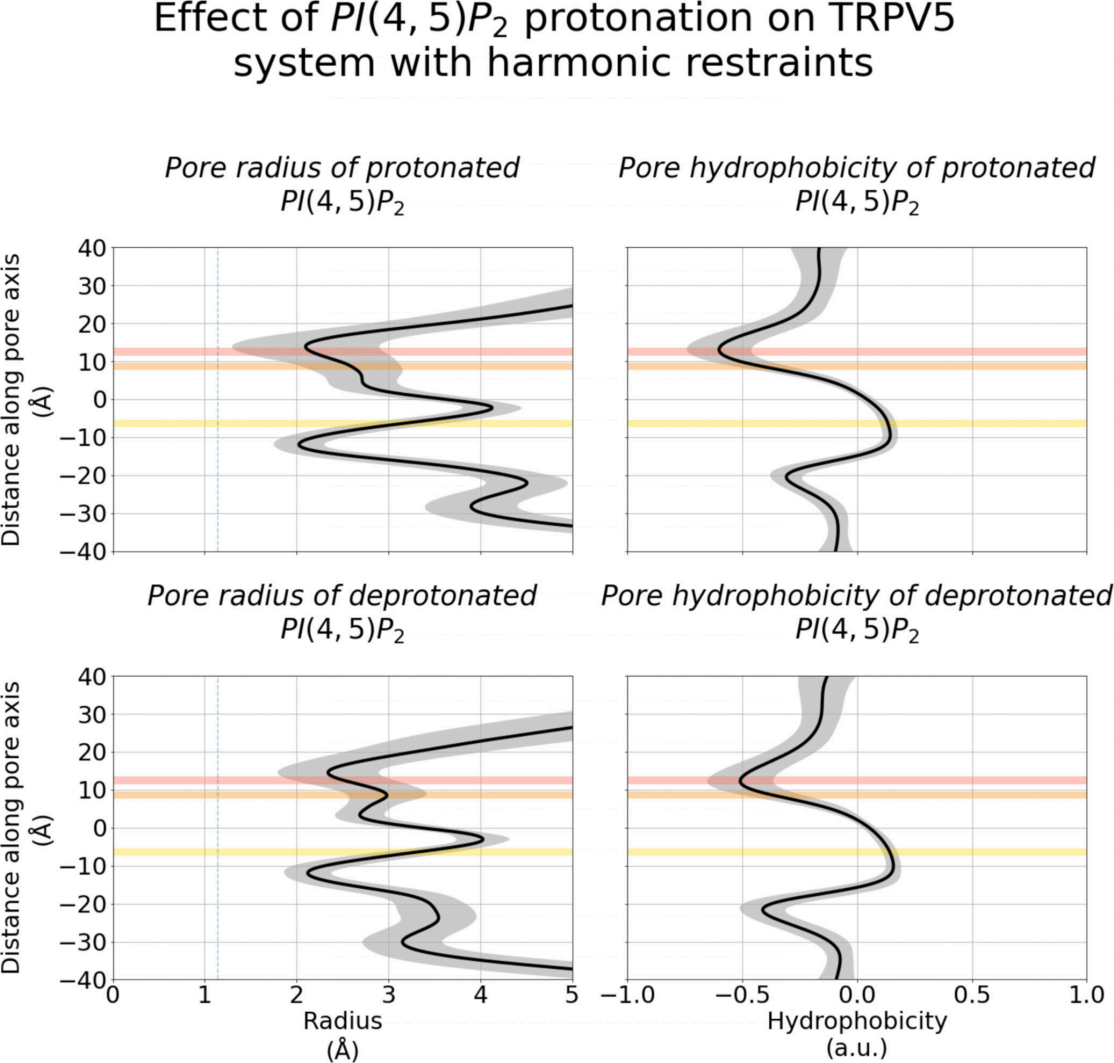
**Pore architecture of TRPV5 channels from MD simulations with protonated (top) and deprotonated (bottom) PI(4,5)P**_**2**_ **molecules.** The plots show the mean radius and hydrophobicity of the channel with respect to the relative *z* coordinate. The shaded gray region represents the SD. The average position of binding sites A, B, and C are shown as shaded red, orange, and yellow regions, respectively. The dashed line represents the radius of a dehydrated Ca^2+ ^ion.

Fig. S1 shows voltage-dependence of occupancy and ion selectivity. Fig. S2 shows additional cation binding sites in TRPV5. Fig. S3 shows pore radii and hydrophobicity in simulations of TRPV2, TRPV3, TRPV5, and TRPV6. Fig. S4 shows occupancy and residence times at postulated W583 ion binding site. Fig. S5 shows permeation traces at low ion concentration. Fig. S6 shows permeation traces in low voltage simulations. Fig. S7 shows impact of binding site affinity differences on exSSI. Fig. S8 shows total correlation of cation permeation between all binding sites in TRPV2, TRPV3, TRPV5, and TRPV6. Fig. S9 shows SF backbone dihedral distributions. Fig. S10 shows multiple sequence alignment of TRPV SF sequences. Fig. S11 shows effect of PI(4,5)P_2_ on pore radii. Tables S1, S2, and S3 show MD simulation details. Table S4 shows permeation times. Table S5 shows SSI details. Table S6 shows selectivity in dicationic solutions. Table S7 shows root-mean-square fluctuation (RMSF) of the SF backbone. Supplemental text at the end of the PDF provides additional information.

## Results

### Continuous permeation of Ca^2+^ and Na^+^ in open-state TRPV5 and TRPV6 channels

We performed MD simulations of the pore domain of open-state TRPV5 (Hughes et al., 2018) and TRPV6 (McGoldrick et al., 2018) channels embedded in 1-palmitoyl-2-oleoyl-sn-glycerol-3-phosphocholine lipid bilayers under transmembrane voltage (approximately −410 mV). The aqueous solutions contained either 150 mM CaCl_2_ or 150 mM NaCl (herein referred to as monocationic solutions) or a mixture consisting of 75 mM CaCl_2_ and 75 mM NaCl (herein referred to as dicationic solutions). All simulations performed with Ca^2+^ ions utilized the multisite Ca^2+^ model developed by Zhang et al. (2020) unless otherwise stated. In both the monocationic and the dicationic solutions, the applied voltage drove a continuous flow of permeating ions through all investigated open-state TRPV channels. Overall, we recorded 433 complete inward channel crossings for Ca^2+^ and 417 for Na^+^ in simulations of the Ca^2+^-selective TRPV channels.

In TRPV5 and TRPV6, Ca^2+^ ions traversed the entire pore within average time spans of 28.4 ± 3.9 ns (TRPV5) and 12.0 ± 1.0 ns (TRPV6; Table S4). The calculated Ca^2+^ and Na^+^ conductances from our simulations are shown in Table 3. The considerable Na^+^ conductances we observed agree with the experimental finding that the highly Ca^2+^-selective TRPV channels conduct Na^+^ well in the absence of Ca^2+^ (Nilius et al., 2000; Vennekens et al., 2000; Voets et al., 2003; Bödding and Flockerzi, 2004). Notably, these conductances are in quantitative agreement with published values measured for Na^+^ in vitro (Yue et al., 2001; Cha et al., 2007). By contrast, control simulations of TRPV5 using the default CHARMM36m force field parameters for Ca^2+^, but otherwise identical conditions, did not exhibit ion permeation; instead, the Ca^2+^ ions remained tightly bound to the protein ion binding sites for the entire course of the simulations. This observation is reflective of the shortcomings of standard parameters for divalent cations in fixed-point charge force fields and highlights the improved accuracy of multisite Ca^2+^ models in simulating divalent cation permeation and reproducing in vitro conductances. In addition, no Cl^−^ anions were observed to permeate TRPV channels in any of our simulations.

**Table 3.** Calculated conductances from MD simulations of ion permeation in Ca^2+^-selective TRPV channels

	Conductance (pS)			
	Ca^2+^	Na^+^	Ca^2+^ and Na^+^	Experimental literature values
TRPV5	53 ± 7	49 ± 6	92 ± 10	59 ± 6 (Na^+^) (Cha et al., 2007)
TRPV6	117 ± 12	61 ± 6	29 ± 6	58 ± 4 (Na^+^) (Yue et al., 2001)

Mean inward conductances and SEM were calculated from overlapping 50 ns windows from fivefold replicated 250 ns simulations. Permeation of monocationic Ca^2+^ or Na^+^ cations was simulated in a solution of 150 mM CaCl_2_ or 150 mM NaCl, respectively. Permeation of a dicationic mixture of Ca^2+^ and Na^+^ cations was investigated in a solution of 75 mM CaCl_2_ and 75 mM NaCl.

We note that the *P*_*Ca*_*/P*_*Na*_ values obtained from our simulations overall show lower Ca^2+^ selectivity than the reported literature values (Table S6). We surmised that this might be, at least partially, due to the higher voltages used in our simulations to enhance the sampling rate. Supplementary simulations performed at a lower voltage demonstrated that, indeed, the selectivity for Ca^2+^ increases with lower voltages across the membrane (Fig. S1). Below a voltage threshold of *∼*205 mV, however, the sampling of permeation events in the simulations became very poor, such that we were not able to reliably probe the precise voltage range used in the experiments.

We can, additionally, not rule out the contribution of force field inaccuracies. Our simulations with Na^+^ show a remarkable agreement between experimentally recorded and simulated conductance. Even though the Ca^2+^ model we used has been carefully parameterized (Zhang et al., 2020), modeling divalent cations is a far from trivial task, and this is the first multisite Ca^2+^ model with which simulations of ion channel current have become possible. It can therefore not be excluded that further iterations of model refinement may eventually be required to not only reflect experimental solvation-free energies and protein affinity (Zhang et al., 2020) but also accurately reproduce experimental conductance values. This includes kinetic factors such as the correct characteristics of ion (electro-)diffusion in bulk solvent.

### Pore cation binding sites and their preference for Ca^2+^ binding

Prior to the determination of the atomic structures of Ca^2+^ channels and the development of channel-permeable models for Ca^2+^ ions, it had been suggested from experimental observations that Ca^2+^ channels may obtain their selectivity through competition, i.e., by divalent cations, such as Ca^2+^, binding more tightly to their ion binding sites than monovalent cations, such as Na^+^ (Corry et al., 2001; Hille, 2001).

**Figure 2.**
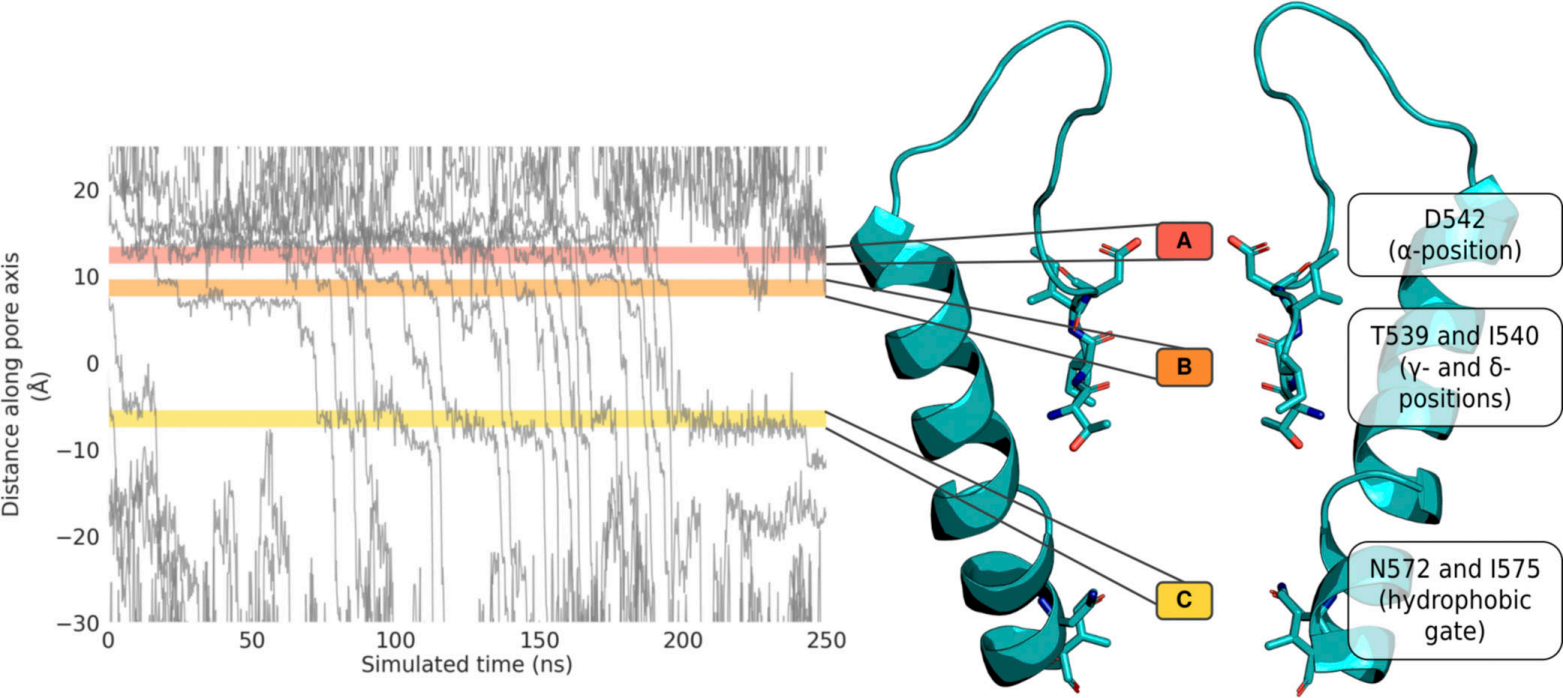
**Schematic of cation binding sites identified in the Ca**^**2+**^**-selective TRPV5 channel.** Permeation traces of the *z*-coordinate of permeating Ca^2+^ cations over time established that cations are bound at three regions within the pore (left). The location of the residues constituting these three binding sites in Ca^2+^-selective TRPV channels is shown on the structure of TRPV5 (right). Please note, only cations that fully permeate through the pore within the 250 ns simulation are shown in the plot (left).

By analyzing the individual traces of permeating Ca^2+^ cations along the pore axis *z* of TRPV5 and TRPV6 over time, we identified three cation-binding sites inside the channels (Fig. 2). We refer to these cation binding sites as sites A, B, and C, viewed from the extracellular entrance of the channel SF to the hydrophobic lower gate. The three cation binding sites were further confirmed by 3-D density analysis using PENSA (Vögele et al., 2021; Vögele et al., 2022; Fig. S2). The PENSA analysis also identified a number of cation-binding sites outside of the pore within the extracellular loops of both TRPV5 and TRPV6 (Fig. S2), in line with the previous suggestion that TRPV6 contains negatively charged “recruitment sites” that funnel cations toward the entrance of the pore (Saotome et al., 2016; Sakipov et al., 2018).

Of the three binding sites we observed, binding site A is formed by the carboxylate oxygen atoms of the ring of acidic residues at the SF entrance (referred to here as the SF *α*-position); binding site B is formed by the carbonyl oxygen atoms of the bottom two SF residues (SF *γ*- and *δ*-positions); and binding site C is formed jointly by the hydrophobic gate consisting of a ring of isoleucine residues (I575 in TRPV5) and the amide oxygen atoms of the neighboring asparagine residues (N572 in TRPV5) near the cytoplasmic exit of the pore (Fig. 2). The location of these binding sites coincides with constrictions in the pore profile, as determined using CHAP (Klesse et al, 2019; Fig. S3; see Fig. S11). The distance between binding sites A and B is ∼5 Å, and that between binding sites B and C is ∼14 Å. We note that Hughes et al. (2018) reported a further constriction below the hydrophobic gate (binding site C) formed by W583 in the TRPV5 structure, and an analogous constriction at W583 can be observed in TRPV6. However, our simulations do not suggest that the side chains of W583 constitute a functionally important ion binding site, as shown in Fig. S4.

**Figure 3.**
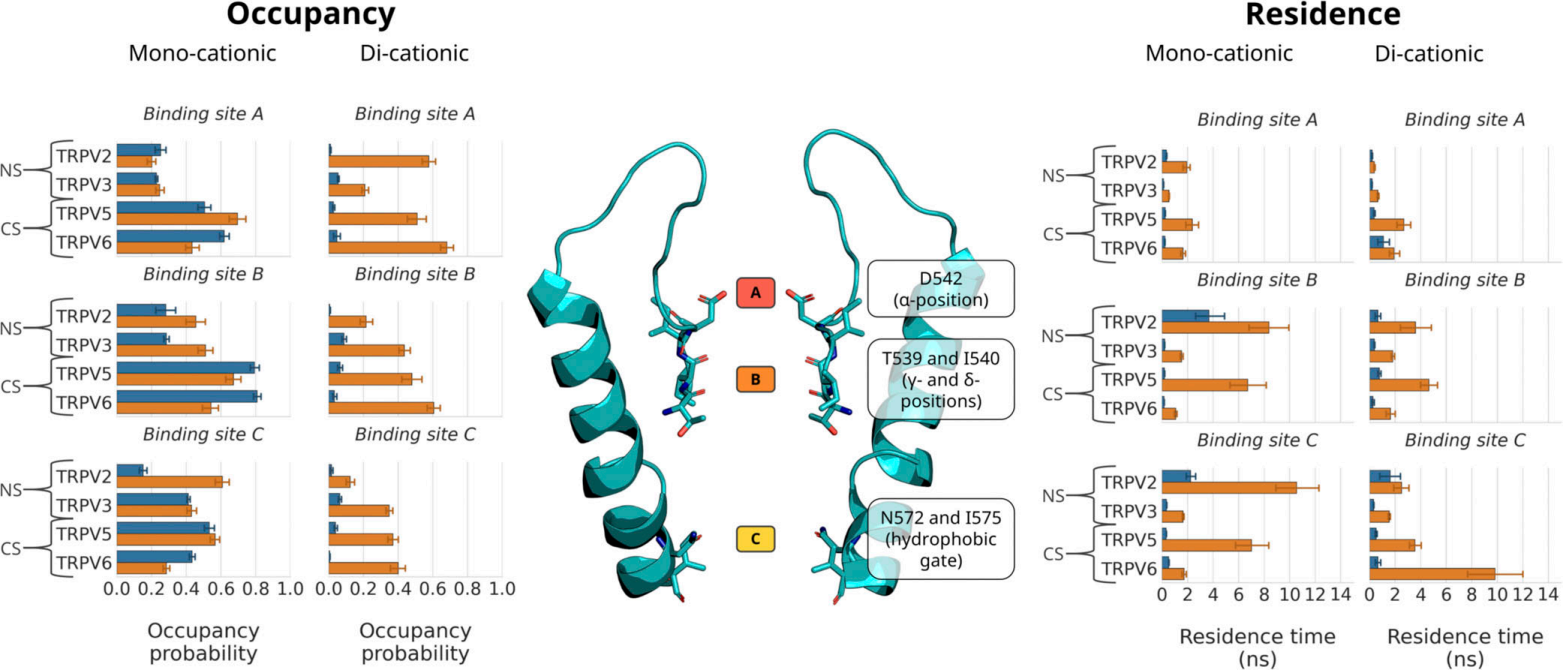
**Occupancy probability (left) and residence times (right) of Ca**^**2+**^ **(orange columns) and Na**^**+**^ **(blue columns) cations from simulations of ion permeation in both monocationic and dicationic ion solutions.** The plots show the occupancy probability of all proteins in this study, including Ca^2+^-selective (CS) and non-selective (NS) TRPV channels. The mean occupancy probability and SEM were calculated from non-overlapping 50 ns windows from fivefold replicated 250 ns simulations, the mean residence time and SEM from fivefold replicated 250 ns simulations. The location of the residues constituting binding sites A, B, and C in Ca^2+^-selective TRPV channels is shown in the structure of TRPV5 (center).

In monocationic Ca^2+^ solutions, the three binding sites showed Ca^2+^ occupancy probabilities of 0.69 ± 0.05, 0.67 ± 0.04, and 0.57 ± 0.03 in TRPV5, and 0.43 ± 0.04, 0.54 ± 0.05, and 0.29 ± 0.02 in TRPV6 (from A to C, respectively; Fig. 3). In monocationic Na ^+^ solutions, similar occupancies were observed. However, the Na^+^ residence times (*t*_*r*_) at the three binding sites were markedly lower than those observed for Ca^2+^, with ratios of *t*_*r*_ (Ca^2+^):*t*_*r*_ (Na^+^) varying between ∼35:1 and ∼3:1 (Fig. 3). These residence times suggest that Ca^2+^ ions have a greater affinity for these binding sites than Na^+^.

This observation was further substantiated when the occupancy of binding sites in dicationic solutions was analyzed, in which Ca^2+^ and Na^+^ cations are competing for the binding sites. Binding sites A, B, and C in the Ca^2+^-selective channels all showed high occupancies with Ca^2+^ in the mixed cationic solutions (Fig. 3). Across all the replicate simulations, we recorded average Ca^2+^ occupancies of 0.51 ± 0.05, 0.48 ± 0.06, and 0.37 ± 0.03 for binding sites A, B, and C in TRPV5, respectively, and of 0.68 ± 0.04, 0.61 ± 0.04, and 0.40 ± 0.04 for binding sites A, B, and C in TRPV6 (Fig. 3). By contrast, the Na^+^ occupancy of each of the three binding sites under these conditions was found to be below 0.07, both in TRPV5 and TRPV6; that is, the ratio between Ca^2+^ and Na^+^ occupancy varies between ∼85:1 and ∼7:1 (Fig. 3). These values indicate a free energy difference of between 11.5 and 5.0 kJ mol^−1^ for the preferential binding of Ca^2+^ over Na^+^.

### A highly cooperative knock-on mechanism between three cation binding sites underpins selective Ca^2+^ permeation in TRPV channels

The observed increased affinity for Ca^2+^ cations at the pore binding sites compared to Na^+^ means that, in a mixed solution, Ca^2+^ will preferentially occupy these binding sites; however, this also implies that Ca^2+^ ions face a greater energy barrier when they dissociate from the binding sites. In monocationic solutions, this would result in a greatly reduced Ca^2+^ conductance with respect to Na^+^. For instance, based on our observed residence times in monocationic solutions, we would expect an ∼12-fold reduced Ca^2+^ unbinding rate compared to Na^+^ for binding site A in TRPV5. However, a much-reduced Ca^2+^ conductance is neither observed in our simulations nor the experimental literature. Due to the divalent charge of Ca^2+^, increasing the affinity to cation binding sites, this dichotomy had previously been suggested to exist, and it was hypothesized that this paradox could be resolved by assuming cooperativity between successive unbinding events such as in a knock-on mechanism (Corry et al., 2001; Hille, 2001).

In the classic knock-on mechanism, which for example underpins K^+^ channel function, ions transition into and out of multiple ion-binding sites in a highly correlated fashion (Armstrong, 1971; Hille and Schwarz, 1978; Neyton and Miller, 1988; Morais-Cabral et al., 2001; Köpfer et al., 2014). For example, early experiments by Hodgkin and Keynes and later flux-ratio measurements established that 3–3.4 K^+^ ions moved in lockstep with each other during permeation through K^+^ channels (Hodgkin and Keynes, 1955; Stampe and Begenisich, 1996).

**Figure 4.**
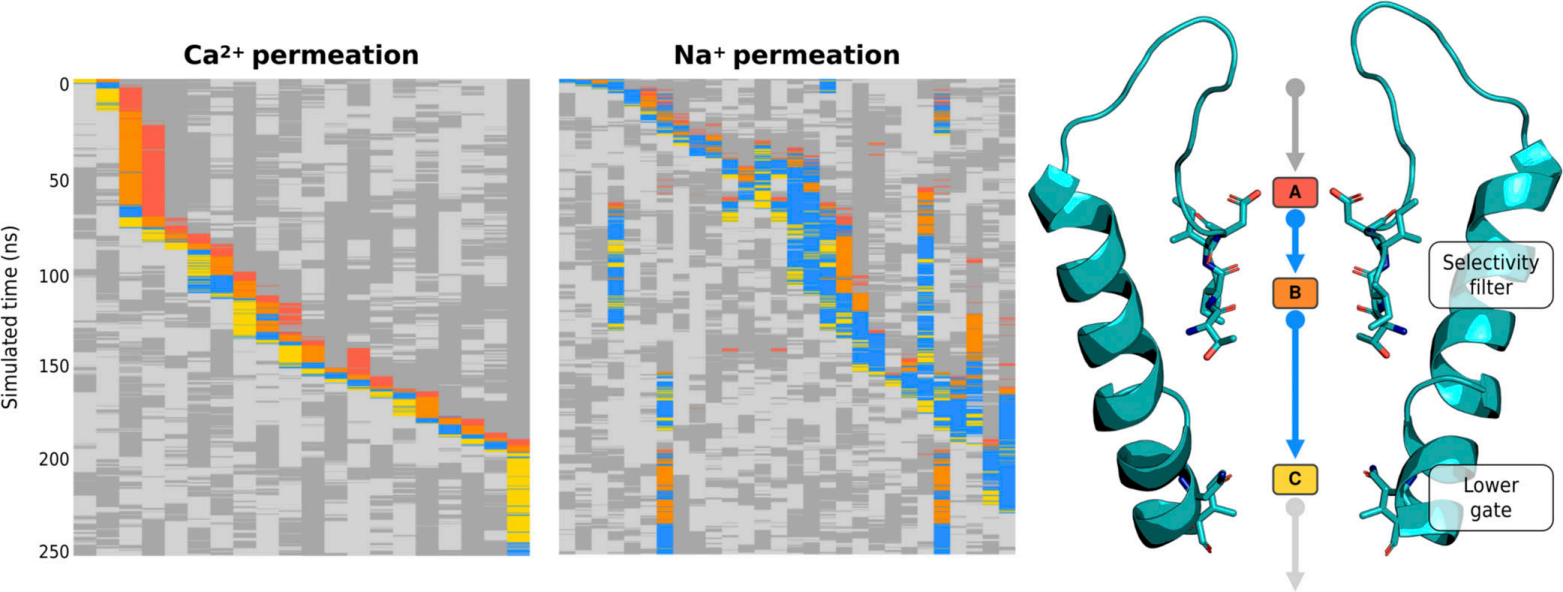
**Permeation state plots of permeating Ca**^**2+**^ **(left) and Na**^**+**^ **(center) cations through the Ca**^**2+**^**-selective TRPV5.** Permeation state plots show the state of each permeating cation (columns) at a given time point (rows) by assigning a state to the ions to indicate whether the cation is bound to a binding site, transitioning between binding sites, or in the bulk solution: bound to A, red; bound to B, orange; bound to C, yellow; transiting within the pore but not bound to a binding site, blue; located inextracellular solvent, dark gray; located in intracellular solvent, light gray. Comparison of permeation state plots for Ca^2+^ and Na^+^ cations shows that Ca^2+^ permeation proceeds in a well-ordered manner with three Ca^2+^ cations within the pore and knocking adjacent cations to the next binding site. Na^+^ permeation, on the other hand, is far less ordered, with regularly more than three Na^+^ cations within the pore at a given time. Each plot (left and center) shows exemplars from a single 250 ns simulation of TRPV5 performed in monocationic 150 mM CaCl_2_ or 150 mM NaCl, respectively. The structure of TRPV5 shows the colors used in the permeation state plots and the location of the residues constituting the three binding sites (right). Please note, only cations that fully permeate through the pore within the 250 ns simulation are shown in the plots, whereas the binding site occupancies reported above reflect both permeating and non-permeating ions (left and right).

For each permeating ion in a simulation of TRPV5, Fig. 4 shows the association and dissociation of Ca^2+^ and Na^+^ ions at binding sites A, B, and C from top to bottom as color code (bound to A, red; bound to B, orange; bound to C, yellow; transiting within the pore but not bound to a binding site, blue; located in extracellular solvent, dark gray; and located in intracellular solvent, light gray). As can be seen for Ca^2+^ in TRPV5 for example (Fig. 4 left), the plot demonstrates that (i) permeating Ca^2+^ ions spend the vast majority of their time within the pore at the three binding sites (reflected in the scarcity of blue boxes vs. red, orange, and yellow), (ii) dual and triple occupancy of the three sites, A, B, and C, with Ca^2+^ frequently observed (horizontal slices across plot: triple occupancy is observed in 27.2% of the simulation frames, dual occupancy in 49.7%), and (iii) transitions between states show a high degree of correlation, i.e., the ions frequently move in concert into and out of their respective binding sites (horizontal slices; binding state transitions). By contrast, during Na^+^ permeation (Fig. 4 center), the ions are predominantly transiting across the pore without occupying particular binding sites for extended time spans (blue, on average 53% of the traversal time for each ion).

To go beyond visual inspection of the trajectories and to assess the cooperativity of ion permeation in a quantitative way, we developed a new approach based on mutual information, taking into account the “state” of each ion binding site. To achieve this, we assigned a specific binding state (unoccupied or occupied with a specific ion) to binding sites A, B, and C and used our recently developed approach, SSI (Thomson et al., 2021 *Preprint*), on pairs of adjacent sites to quantify the degree of coupling between ion binding transitions at each of these sites (see Materials and methods). This analysis yields a coefficient quantifying the cooperativity between ion binding and unbinding at neighboring or more distant binding sites, where a greater coefficient signifies a higher degree of coupling. This coupling suggests that when an ion transitions from one site it is more likely that there is a transition at the other. To correct for the non-zero mutual information that samples of two completely independent variables can display due to finite-size effects, we followed the approach of McClendon et al. (2009) and Pethel and Hahs (2014) to yield excess mutual information, or *exSSI*. We also determined a theoretical upper limit for the maximum mutual information that can be shared between two binding sites by using the minimum state entropy among the two sites. Note that this quantity represents an absolute upper limit; reaching it would require both binding sites to exhibit idealized simultaneous states and state transitions throughout the entire simulated time.

**Figure 5.**
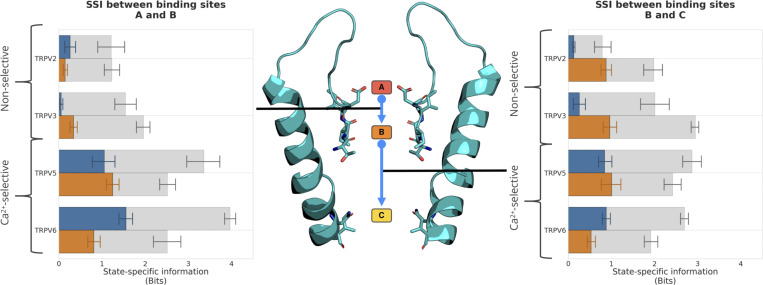
***exSSI* between ion binding sites quantifies the degree of cooperativity in the knock-on mechanism of cation permeation in TRPV channels.** The mean *exSSI* and SEM between transitions from binding sites A and B (left) and binding sites B and C (right) are shown for Ca^2+^ (orange) and Na^+^ (blue) cations in monocationic solutions. For each *exSSI*, the mean maximum *exSSI*_*max*_ and standard error are also shown (gray), and the *exSSI*_*norm*_ is reported in Table S5.

The SSI analysis showed that in the Ca^2+^-selective TRPV channels under the simulated conditions, TRPV5 and TRPV6, a high level of information above noise is shared between the transition of ions into and out of binding sites A and B, respectively, both for the permeation of Ca^2+^ and Na^+^ (*exSSI* between 0.8 and 1.6 bits; Table S5 and Fig. 5). This suggests that the ion binding and unbinding processes at each of these binding sites are coupled to one another, constituting a knock-on mechanism at relatively short range. We observed three to four water molecules on average between cations bound at binding sites A and B during knock-on, demonstrating a “soft” knock-on mechanism to be in place, as opposed to the “direct” knock-on mechanism between dehydrated K^+^ ions in K^+^-selective cation channels (Köpfer et al., 2014). As detailed further below, our simulations indicate that only a moderate level of ion desolvation occurs in the SF of the studied TRPV channels, such that the hydration shell of the permeating ions remains largely intact during knock-on.

Similarly, the transitions of ions into and out of binding sites B and C show a large degree of correlation for both Ca^2+^ and Na^+^ (Fig. 5). In the case of binding sites B and C, however, this requires a remote knock-on mechanism to be in operation, since these sites are ∼14 Å apart. The concept of a remote knock-on event was first proposed by Tindjong et al. (2013) based upon Brownian dynamics simulations and observed by Zhang et al. (2020) in atomistic MD simulations of Ca^2+^ permeation in the RyR1 channel. Our SSI analysis suggests that the degree of cooperativity in the remote knock-on mechanism between binding sites B and C (Fig. 5 right) is slightly smaller than the cooperativity in the closer knock-on mechanism between binding sites A and B (Fig. 5 left). Simulations conducted at a lower CaCl_2_ concentration (Fig. S5) and reduced transmembrane voltage (Fig. S6) confirmed that the knock-on mechanism observed occurs also under these milder conditions; however, we did not achieve enough sampling to recalculate the *exSSI* values at these conditions.

### Cation permeation in non-selective TRPV channels shows a lower degree of cooperativity

To determine if the remaining, non-selective TRPV channels showed a different permeation mechanism, we next performed simulations of the open-state TRPV2 (Dosey et al., 2019) and TRPV3 (Singh et al., 2019) channels using the same simulation approach as described for the Ca^2+^-selective TRPV channels. These simulations of non-selective TRPV channels also showed continuous ion permeation, with cation conductances, again, in good agreement with published conductance values measured in vitro (Table 4). Overall, we recorded 706 complete inward channel crossings for Ca^2+^ and 1,176 for Na^+^ from simulations of the non-selective TRPV channels.

**Table 4.** Calculated conductances from MD simulations of ion permeation in non-selective TRPV channels

	Conductance (pS)			
	Ca^2+^	Na^+^	Ca^2+^ & Na^+^	Experimental literature values
TRPV2	40 ± 4	18 ± 3	43 ± 7	28 (Na^+^) (Zhang et al., 2016)
TRPV3	196 ± 10	299 ± 10	222 ± 13	201 (Na^+^) (Chung et al., 2004)

Mean inward conductances and SEM were calculated from overlapping 50 ns windows from fivefold replicated 250 ns simulations. Monocationic solutions at 150 mM concentration; dicationic mixture of Ca^2+^ and Na^+^ at a concentration of 75 mM each.

The occupancy of binding sites B and C in the TRPV2 and TRPV3 systems showed no clear difference to the Ca^2+^-selective channels. By contrast, the occupancy of binding site A was reduced by ∼50% for both Na^+^ and Ca^2+^ ions in the monocationic solutions (Fig. 3). This suggests that cations are less well coordinated at binding site A in the non-selective TRPV channels, leading to lower affinity binding in the monocationic solutions. All binding sites, however, exhibited a preference for binding Ca^2+^ in the dicationic solutions. Note that, in the non-selective channels TRPV2 and TRPV3, this is coupled with a particularly low residence time for Ca^2+^ at binding site A, again suggesting higher exchange rates and weaker binding, despite its occupancy. That is, many Ca^2+^ ions are observed to diffuse back from A to bulk solution in TRPV2 and TRPV3.

We were therefore curious if the three-site knock-on mechanism described previously for Ca^2+^-selective TRPV channels is also at play in the non-selective TRPV channels. Our SSI analysis confirmed that the cooperativity between binding sites B and C in the non-selective TRPV channels was comparable with those calculated for the Ca^2+^-selective TRPV channels (Table S5 and Fig. 5). However, the correlation between ion binding transitions at binding sites A and B was substantially reduced in both of the non-selective TRPV systems (Fig. 5). The nearly complete absence of cooperativity from binding sites A and B demonstrates that a knock-on mechanism is not occurring between these two sites in the non-selective TRPV channels. Instead, our findings suggest that cation permeation in the non-selective TRPV channels occurs via a two-site knock-on mechanism between binding sites B and C.

Since the ion occupancy observed at binding site A is reduced in the case of the non-selective TRPV channels, it is plausible that this lower affinity also impacts the coupling between transitions at binding sites A and B. To test this notion further, the relationship between affinity differences of a pair of binding sites and the knock-on co-operativity was tested systematically by using a toy model with two energy wells (binding sites) possessing a range of different depths (affinities). As shown in Fig. S7, there is a linear relationship between the affinity difference and the observed *exSSI*. This demonstrates that the diminished affinity of binding site A is likely to be the major reason for the loss of cooperativity in the SF of the non-selective TRPV channels. Our results show that similar binding affinity is a necessary but not sufficient condition for a high degree of cooperativity between two cation binding sites.

Based on our SSI approach to quantify mutual information in binding and unbinding events at different ion binding sites and using the concept of total correlation to evaluate the overall cooperativity in a system across all coupled events, we next calculated the total correlation of ion permeation for all the TRP channels investigated. The reduction in the number of correlated knock-on sites within the non-selective TRP channels can be distinctly seen when comparing the total correlation for each of the non-selective and Ca^2+^-selective TRPV channels (Fig. S8).

The ion binding sites in non-selective TRPV channels preferentially bind Ca^2+^ over Na^+^, as in Ca^2+^-selective TRPV channels, which explains why the non-selective TRPV channels in fact show slight Ca^2+^ selectivity (*P*_*Ca*_*/P*_*Na*_ ∼10:1; Owsianik et al., 2006). However, our data suggest that the reduced level of coordination at binding site A, and especially the effect on the three-site cooperativity it imparts together with sites B and C, reduces the Ca^2+^ selectivity from *P*_*Ca*_*/P*_*Na*_ ∼100:1 seen in TRPV5 and TRPV6, and in this way ultimately determines the difference between Ca^2+^-selective and non-selective permeation.

### Structural features distinguishing Ca^2+^-selective from non-selective permeation

**Figure 6.**
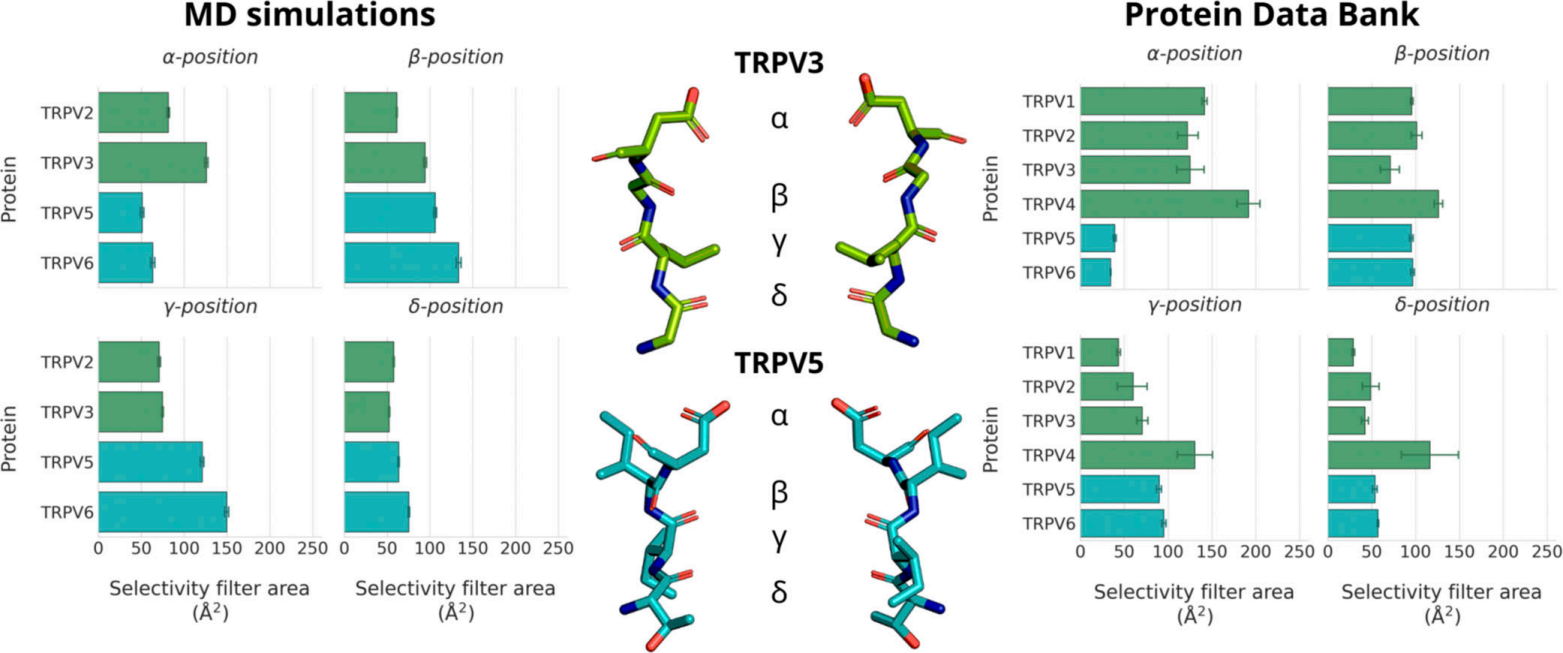
**Architecture of the four-residue selectivity filter of the investigated TRPV channels.** The area formed between the carboxylate oxygen atoms (*α*-position) and backbone carbonyl atoms (*β*-, *γ*-, and *δ*- positions) was calculated for all Ca^2+^-selective and non-selective TRPV systems simulated as part of this study (left), and for all TRPV structures available in the PDB at the time of writing (right). The SF structures of the Ca^2+^-selective TRPV5 and non-selective TRPV3 are shown for reference (center). The mean area between SF residues and SEM was calculated from non-overlapping 50 ns windows from fivefold replicated 250 ns simulations in 150 mM CaCl_2_. The mean area between SF residues and SEM from PDB structures was calculated from all available homotetrameric TRPV structures at the time of writing.

To understand why cation coordination at binding site A is weakened in non-selective TRPV channels, decoupling its cooperativity, we investigated the area formed between the four subunits of the TRPV channel for each residue in the selectivity filter. The cross-sectional area formed by the carboxylate oxygen atoms at the SF *α*-position is larger in the non-selective TRPV channels than in the Ca^2+^-selective TRPV channels (Fig. 6). Interestingly, despite the increased average area formed by the carboxylate oxygen atoms at the *α*-position residue in the selectivity filter, the average area formed by the carbonyl oxygen atoms at both the *γ*- and *δ*-positions are smaller in non-selective TRPV channels than in Ca^2+^-selective TRPV channels (Fig. 6). These differences in selectivity architecture were further confirmed using the pore profile calculated using CHAP (Klesse et al, 2019; Fig. S3). Our simulations showed no differences in the flexibility of selectivity filters between Ca^2+^-selective and non-selective TRPV channels as determined by RMSF calculations of the backbone atoms (Table S7); we did however observe a small difference in the backbone dihedral angle distribution of the *β*-position residue, which we predict to be due to differences in stabilizing hydrophobic interactions of the residue side-chain (Fig. S9, S10, and S11).

To expand the geometric analysis to all available TRPV channel structures, we also calculated the average area formed by selectivity filter residues from all available TRPV structures deposited in the PDB (Fig. 6). Analysis of these static structures showed the same two trends observed within our MD simulations: (1) Ca^2+^-selective TRPV channels clearly have a smaller average area formed by the carboxylate oxygen atoms of the SF *α*-position; and (2) non-selective TRPV channels have a slightly narrower constriction at the SF *γ*- and *δ*-position residues. Our MD simulations suggested that the wider opening at the *α*-position leads to a weaker cation binding interaction at binding site A and cation coordination, which, in turn, decouples binding site A from the co-operative knock-on mechanism that underpins permeation in the Ca^2+^-selective TRPV channels.

The non-selective channels possess a narrower constriction formed by the side chains and carbonyl groups of the *γ*- and *δ*-position residues forming binding site B, whereas the Ca^2+^-selective TRPV channels exhibit an even narrower constriction at binding site A. Our simulations suggest that the greater occupancy of binding site A is a result of the more confined geometry of this charged binding site in the Ca^2+^-selective TRPV channels. This, in turn, leads to a higher occupancy of the uncharged binding site B in the Ca^2+^-selective TRPV channels (see Fig. 3), as binding site B receives cations from the adjacent binding site A via the knock-on mechanism, rather than having to bind them from bulk solution, as would be the case in the non-selective channels. Thus, despite the constriction not being as narrow, binding site B has a higher occupancy in the Ca^2+^-selective than in the non-selective TRPV channels.

### Ca^2+^-selective permeation is not strongly linked to the solvation states of permeating cations

A previously reported mechanism of how cation selectivity can be achieved is by desolvation of permeating cations. Differences in desolvation energies between cationic species provide a thermodynamic penalty that can be more favorable for the permeation of one cationic species over another. Such a mechanism has been reported to underpin K^+^ selectivity over Na^+^ in K^+^ channels (Köpfer et al., 2014; Kopec et al., 2018), for example. Alternative mechanisms suggested to yield K^+^ selectivity are based on protein flexibility, especially the plasticity of the K^+^ channel SF (Noskov et al., 2004), the number of stacked ion binding sites in the SF (Derebe et al., 2011), where a reduction from four to three has been shown to abolish K^+^ selectivity, the kinetic model of selectivity (Thompson et al., 2009; Kim and Allen, 2011), and the coordination model (Bostick and Brooks, 2007; Varma et al., 2008). By contrast, Na^+^ selectivity has been suggested to rely chiefly on a “snug fit” coordination of the Na^+^ ion in the SFs of eukaryotic Na_V_ channels and the preservation of its solvation shell while permeating bacterial Na_V_ channels (Dudev and Lim, 2014).

**Figure 7.**
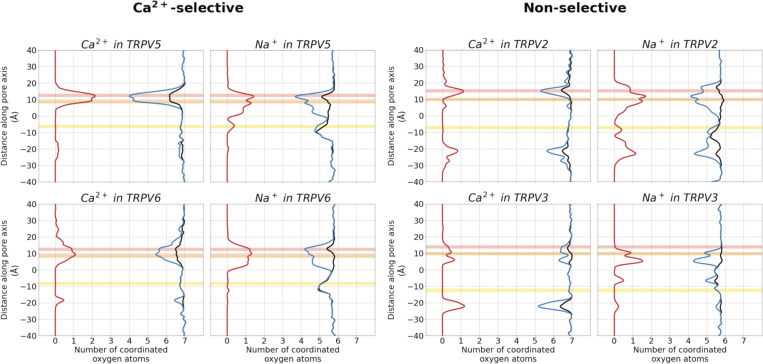
**Solvation state of permeating cations as they permeate through TRPV channels.** The mean number of oxygen atoms of water molecules (blue), the number of oxygen atoms of protein residues (red), and total number of any oxygen atoms (black) within 3 Å of each permeating cation are plotted. The curves were smoothed using a Gaussian filter with a sigma value of 3.

To investigate whether a high degree of desolvation was a dominant factor in Ca^2+^ selectivity in TRPV channels, we determined the number of oxygen atoms within a 3 Å radius of the cations, representing their first solvation shell (Fig. 7). In the bulk solution, both Ca^2+^ and Na^+^ cations showed their expected water coordination number of 7 and 5.6, respectively. As the cations entered the pore, we saw a small degree of partial dehydration of permeating cations at the SF (Fig. 7). In particular, the carboxylate oxygen atoms of the acidic residue at the entrance of the SF coordinated an incoming cation, with these displacing up to approximately two coordinated water molecules from the first solvation shell of the cation. We also observe some desolvation around, or below, binding site C. However, no major differences in the solvation shell of permeating Ca^2+^ or Na^+^ cations, or indeed between the Ca^2+^-selective and non-selective TRPV channels were observed. The finding that the degree of desolvation does not differ substantially between Ca^2+^-selective and non-selective channels indicates that their selectivity is not exclusively based on a mechanism of ion dehydration. The largest level of desolvation is seen at ion binding site A in the TRPV5 channel for Ca^2+^. Since the overall desolvation penalty for Ca^2+^ is larger than for Na^+^ (Marcus, 1991), one would expect this to lead to the preferential binding of Na^+^ at this site, whereas the opposite is observed (Fig. 3), further arguing against dehydration as a major selectivity mechanism in TRPV channels.

## Discussion

Our simulations showed three main cation binding sites in the permeation pathway of TRPV channels, which we term binding sites A, B, and C. Binding sites A and B are formed by the carboxylate oxygen atoms of the *α*-position residue of the SF and by the carbonyl oxygen atoms of the *γ*- and *δ*-position residues of the SF, respectively. Binding site C is located just above the hydrophobic lower gate of the pore and is formed by the isoleucine residues of the lower gate and amide oxygen atoms of the neighboring asparagine residues.

The identification of these cation-binding sites in our MD simulations is in agreement with previously published TRPV structures and other MD simulations. In their crystal structure of the *Rattus norvegicus* TRPV6, Saotome et al. (2016) identified three regions of electron density within the channel pore which they interpreted as cation-binding sites. It should be noted that there are some minor differences in the residues forming binding site C to the binding site reported here, likely due to the structure of TRPV6 of Saotome et al. (2016) being in the closed-state, rather than the open-state structure of McGoldrick et al. (2018) used in the present study. During the transition from a closed state to the open state, TRP channels undergo a rotation about the pore-forming S6 helix, which changes the pore-facing residues (McGoldrick et al., 2018).

Moreover, numerous structures of TRPV channels deposited within the PDB are resolved with cations bound at one of the cation-binding sites identified in our MD simulations. These include structures from several orthologs of the TRPV channels simulated in this study, as well as of the TRPV1 and TRPV4 channels that were not simulated in this study. For example, structures of the closed-state TRPV1 channel of *R*.* norvegicus* (Kwon et al., 2021) and the open-state TRPV4 channel of *Homo sapiens* in complex with 4*α*-PDD (Botte et al., 2020 *Preprint*), both model cations bound at binding site B. This observation further validates the existence of the identified cation binding sites, as well as their conservation among TRPV channels and perhaps the wider TRP superfamily.

In addition to structural data, Sakipov et al. (2018) performed equilibrium MD simulations of Ca^2+^ movement in the closed-state structure of *R*.* norvegicus* TRPV6 and identified cation-binding sites in the SF (Sakipov et al., 2018). The binding sites from this work are also generally in agreement with our simulation results and the aforementioned structural data. However, Sakipov et al. (2018) reported that their simulation data identified two Ca^2+^ cations residing at binding site A, with adjacent chains each occupying an ion. In accordance with previous x-ray crystallographic data, two ions associated with binding site A are not seen in a major population of our simulation ensembles, which we attribute to the use of an optimized multisite model for Ca^2+^ ions in the present study (Zhang et al., 2020) and increased sampling for improved statistical analysis.

In monocationic solutions, we observed a high probability of at least two of the three binding sites (A, B, and C) being simultaneously occupied with either Na^+^ or Ca^2+^, respectively, with mostly insignificant differences between the occupancy values for Na^+^ and Ca^2+^ at each individual binding site. However, the residence times were markedly reduced at all sites for Na^+^ ions, and overall a tendency toward weaker binding for either ion at binding site A at the extracellular SF entrance was observed. In mixed, dicationic solutions of Na^+^ and Ca^2+^, by contrast, Ca^2+^ ions strongly outcompeted Na^+^ ions for association at all pore-binding sites, both in the Ca^2+^-selective and non-selective TRPV channels. Therefore, according to our data, all the binding sites display a much greater affinity for Ca^2+^. This gave rise to the question of how permeation efficiency for conducting Ca^2+^ ions is achieved in these channels and how this relates to their varying degrees of selectivity since higher affinity binding is usually expected to lead to slower permeation rates.

By ensuring cooperativity between binding/unbinding events at multiple binding sites, permeation rates can be enhanced (Corry et al., 2001; Hille, 2001). We hypothesized that the level of cooperativity between sites A, B, and C in the pore could underpin the difference between highly and less Ca^2+^-selective TRPV channels. We, therefore, developed a novel method to quantify co-operativity during ion permeation in pores with multiple ion binding sites based on mutual information and total correlation measures using the SSI approach (Thomson et al., 2021 *Preprint*). We anticipate that this method will be similarly useful for the study of permeation mechanisms and the basis of selectivity in other channels. Our analysis showed that there is a substantial degree of co-operativity for Ca^2+^ permeation between binding sites B and C across all TRPV channels, whereas a clear distinction exists between the co-operativity between binding sites A and B in Ca^2+^-selective and non-selective TRPV channels. In the non-selective TRPV channels, binding site A is decoupled from binding site B. By contrast, binding sites A and B are even more strongly coupled than binding sites B and C in the case of the Ca^2+^-selective channels. We suggest that this marked difference in co-operativity mechanistically explains the different levels of Ca^2+^ selectivity in TRPV channels.

### Conclusion

We have characterized the cation permeation mechanisms in four members of the TRPV channel family. We identified three cation binding sites within the pore, each of which displayed greater affinity for Ca^2+^ binding over Na^+^. A novel application of mutual information between ion binding and unbinding at consecutive binding sites (SSI) enabled us to quantify the degree of knock-on taking place in ion permeation. This showed that the level of Ca^2+^ selectivity in TRPV channels is determined by the co-operativity, or coupling, between the transitions at three pore cation-binding sites. The Ca^2+^-selective TRPV channels display a highly correlated three-site knock-on, whereas the cation binding site at the extracellular entrance is decoupled from the mechanism in the non-selective TRPV channels, which reduces the overall preference for Ca^2+^ permeation.

## Supplementary Material

Table S1 shows summary of simulation details of Ca^2+^-selective TRPV channels.

Table S2 shows summary of simulation details of non-selective TRPV channels.

Table S3 shows summary of simulation details of additional control simulations of Ca^2+^-selective TRPV channels.

Table S4 shows average time to permeate through the TRPV pore, as defined by the *z* position between binding sites A and C.

Table S5 shows calculated *exSSI* and *exSSI*_*norm*_ values of cation transition from binding sites in MD simulations of TRPV channels.

Table S6 shows selectivity ratios of Ca^2+^ and Na^+^ permeation events from simulations of TRPV channels in a dicationic solution.

Table S7 shows RMSF of the backbone of SF residues of TRPV channels from MD simulations.

## Data Availability

MD simulation inputs and analysis scripts used for this study are deposited in a public GitHub repository, available at: https://github.com/cmives/Ca_selectivity_mechanism_of_TRPV_channels.

## References

[bib103] Abraham, M.J., T. Murtola, R. Schulz, S. Páll, J.C. Smith, B. Hess, and E. Lindahl. 2015. GROMACS: High performance molecular simulations through multi-level parallelism from laptops to supercomputers. SoftwareX. 1:19–25. 10.1016/j.softx.2015.06.001

[bib1] Aksimentiev, A., and K. Schulten. 2005. Imaging alpha-hemolysin with molecular dynamics: Ionic conductance, osmotic permeability, and the electrostatic potential map. Biophys. J. 88:3745–3761. 10.1529/biophysj.104.058727 15764651 PMC1305609

[bib2] Antonides, L.H., Q.W. Hurst, C.M. Ives, K. Ramberg, N. Ostrovitsa, E. Scanlan, M. Caffrey, S.J. Pitt, and U. Zachariae. 2022. The SARS-CoV-2 envelope (E) protein forms a calcium- and voltage-activated calcium channel. bioRxiv. (Preprint posted October 11, 2022). 10.1101/2022.10.11.511775

[bib3] Armstrong, C.M. 1971. Interaction of tetraethylammonium ion derivatives with the potassium channels of giant axons. J. Gen. Physiol. 58:413–437. 10.1085/jgp.58.4.413 5112659 PMC2226036

[bib4] Bagur, R., and G. Hajnóczky. 2017. Intracellular Ca^2+^ sensing: Its role in calcium homeostasis and signaling. Mol. Cell. 66:780–788. 10.1016/j.molcel.2017.05.028 28622523 PMC5657234

[bib5] Berridge, M.J., M.D. Bootman, and H.L. Roderick. 2003. Calcium signalling: Dynamics, homeostasis and remodelling. Nat. Rev. Mol. Cell Biol. 4:517–529. 10.1038/nrm1155 12838335

[bib6] Bödding, M., and V. Flockerzi. 2004. Ca^2+^ dependence of the Ca^2+^-selective TRPV6 channel. J. Biol. Chem. 279:36546–36552. 10.1074/jbc.M404679200 15184369

[bib7] Bostick, D.L., and C.L. Brooks III. 2007. Selectivity in K^+^ channels is due to topological control of the permeant ion’s coordinated state. Proc. Natl. Acad. Sci. USA. 104:9260–9265. 10.1073/pnas.0700554104 17519335 PMC1890482

[bib8] Botte, M., A.K.C. Ulrich, R. Adaixo, D. Gnutt, A. Brockmann, D. Bucher, M. Chami, N. Bocquet, U. Ebbinghaus-Kintscher, V. Puetter, . 2020. Cryo-EM structural studies of the agonist complexed human TRPV4 ion-channel reveals novel structural rearrangements resulting in an open-conformation. bioRxiv. (Preprint posted October 13, 2020). 10.1101/2020.10.13.334797

[bib9] Carafoli, E. 2002. Calcium signaling: A tale for all seasons. Proc. Natl. Acad. Sci. USA. 99:1115–1122. 10.1073/pnas.032427999 11830654 PMC122154

[bib10] Caterina, M.J., M.A. Schumacher, M. Tominaga, T.A. Rosen, J.D. Levine, and D. Julius. 1997. The capsaicin receptor: A heat-activated ion channel in the pain pathway. Nature. 389:816–824. 10.1038/39807 9349813

[bib11] Caterina, M.J., A. Leffler, A.B. Malmberg, W.J. Martin, J. Trafton, K.R. Petersen-Zeitz, M. Koltzenburg, A.I. Basbaum, and D. Julius. 2000. Impaired nociception and pain sensation in mice lacking the capsaicin receptor. Science. 288:306–313. 10.1126/science.288.5464.306 10764638

[bib12] Cha, S.K., W. Jabbar, J. Xie, and C.L. Huang. 2007. Regulation of TRPV5 single-channel activity by intracellular pH. J. Membr. Biol. 220:79–85. 10.1007/s00232-007-9076-2 18004496

[bib13] Chang, Y., G. Schlenstedt, V. Flockerzi, and A. Beck. 2010. Properties of the intracellular transient receptor potential (TRP) channel in yeast, Yvc1. FEBS Lett. 584:2028–2032. 10.1016/j.febslet.2009.12.035 20035756

[bib14] Chavan, T.S., R.C. Cheng, T. Jiang, I.I. Mathews, R.A. Stein, A. Koehl, H.S. Mchaourab, E. Tajkhorshid, and M. Maduke. 2020. A CLC-ec1 mutant reveals global conformational change and suggests a unifying mechanism for the CLC Cl^−^/H^+^ transport cycle. Elife. 9:e53479. 10.7554/eLife.53479 32310757 PMC7253180

[bib15] Chung, M.-K., H. Lee, A. Mizuno, M. Suzuki, and M.J. Caterina. 2004. 2-aminoethoxydiphenyl borate activates and sensitizes the heat-gated ion channel TRPV3. J. Neurosci. 24:5177–5182. 10.1523/JNEUROSCI.0934-04.2004 15175387 PMC6729202

[bib16] Clapham, D.E. 2007. Calcium signaling. Cell. 131:1047–1058. 10.1016/j.cell.2007.11.028 18083096

[bib17] Corry, B., T.W. Allen, S. Kuyucak, and S.-H. Chung. 2001. Mechanisms of permeation and selectivity in calcium channels. Biophys. J. 80:195–214. 10.1016/S0006-3495(01)76007-9 11159395 PMC1301226

[bib18] Darden, T., D. York, and L. Pedersen. 1993. Particle mesh Ewald: An N·log(N) method for Ewald sums in large systems. J. Chem. Phys. 98:10089–10092. 10.1063/1.464397

[bib19] Deng, H., G. Chen, W. Yang, and J.J. Yang. 2006. Predicting calcium-binding sites in proteins - a graph theory and geometry approach. Proteins. 64:34–42. 10.1002/prot.20973 16617426

[bib20] Derebe, M.G., D.B. Sauer, W. Zeng, A. Alam, N. Shi, and Y. Jiang. 2011. Tuning the ion selectivity of tetrameric cation channels by changing the number of ion binding sites. Proc. Natl. Acad. Sci. USA. 108:598–602. 10.1073/pnas.1013636108 21187421 PMC3021048

[bib21] Dosey, T.L., Z. Wang, G. Fan, Z. Zhang, I.I. Serysheva, W. Chiu, and T.G. Wensel. 2019. Structures of TRPV2 in distinct conformations provide insight into role of the pore turret. Nat. Struct. Mol. Biol. 26:40–49. 10.1038/s41594-018-0168-8 30598551 PMC6458597

[bib22] Dudev, T., and C. Lim. 2014. Ion selectivity strategies of sodium channel selectivity filters. Acc. Chem. Res. 47:3580–3587. 10.1021/ar5002878 25343535

[bib23] Evans, D.J., and B.L. Holian. 1985. The Nose-Hoover thermostat. J. Chem. Phys. 83:4069–4074. 10.1063/1.449071

[bib24] Flores-Aldama, L., M.W. Vandewege, K. Zavala, C.K. Colenso, W. Gonzalez, S.E. Brauchi, and J.C. Opazo. 2020. Evolutionary analyses reveal independent origins of gene repertoires and structural motifs associated to fast inactivation in calcium-selective TRPV channels. Sci. Rep. 10:8684. 10.1038/s41598-020-65679-6 32457384 PMC7250927

[bib25] Gowers, R., M. Linke, J. Barnoud, T. Reddy, M. Melo, S. Seyler, J. Domański, D. Dotson, S. Buchoux, I. Kenney, and O. Beckstein. 2016. MDAnalysis: A python package for the rapid analysis of molecular dynamics simulations. Proc.15th Python Sci. conf. 98–105.

[bib26] Hess, B., H. Bekker, H.J. Berendsen, and J.G. Fraaije. 1997. LINCS: A linear constraint solver for molecular simulations. J. Comput. Chem. 18:1463–1472. 10.1002/(SICI)1096-987X(199709)18:12<1463::AID-JCC4>3.0.CO;2-H

[bib27] Hille, B. 2001. Ion Channels of Excitable Membranes. Third edition. Sinauer, Sunderland, MA.

[bib28] Hille, B., and W. Schwarz. 1978. Potassium channels as multi-ion single-file pores. J. Gen. Physiol. 72:409–442. 10.1085/jgp.72.4.409 722275 PMC2228548

[bib29] Hilton, J.K., M. Kim, and W.D. Van Horn. 2019. Structural and evolutionary insights point to allosteric regulation of TRP ion channels. Acc. Chem. Res. 52:1643–1652. 10.1021/acs.accounts.9b00075 31149807 PMC8628317

[bib30] Himmel, N.J., T.R. Gray, and D.N. Cox. 2020. Phylogenetics identifies two eumetazoan TRPM clades and an eighth TRP family, TRP soromelastatin (TRPS). Mol. Biol. Evol. 37:2034–2044. 10.1093/molbev/msaa065 32159767 PMC7306681

[bib31] Hoenderop, J.G.J., R. Vennekens, D. Müller, J. Prenen, G. Droogmans, R.J.M. Bindels, and B. Nilius. 2001. Function and expression of the epithelial Ca^2+^ channel family: comparison of mammalian ECaC1 and 2. J. Physiol. 537:747–761. 10.1113/jphysiol.2001.012917 11744752 PMC2278984

[bib32] Hodgkin, A.L., and R.D. Keynes. 1955. The potassium permeability of a giant nerve fibre. J. Physiol. 128:61–88. 10.1113/jphysiol.1955.sp005291 PMC1365755 14368575

[bib33] Huang, J., S. Rauscher, G. Nawrocki, T. Ran, M. Feig, B.L. de Groot, H. Grubmüller, and A.D. MacKerell Jr. 2017. CHARMM36m: An improved force field for folded and intrinsically disordered proteins. Nat. Methods. 14:71–73. 10.1038/nmeth.4067 27819658 PMC5199616

[bib34] Hughes, T.E.T., R.A. Pumroy, A.T. Yazici, M.A. Kasimova, E.C. Fluck, K.W. Huynh, A. Samanta, S.K. Molugu, Z.H. Zhou, V. Carnevale, . 2018. Structural insights on TRPV5 gating by endogenous modulators. Nat. Commun. 9:4198–4209. 10.1038/s41467-018-06753-6 30305626 PMC6179994

[bib35] Hunter, J.D. 2007. Matplotlib: A 2D graphics environment. Comput. Sci. Eng. 9:99–104. 10.1109/MCSE.2007.55

[bib36] Jo, S., T. Kim, and W. Im. 2007. Automated builder and database of protein/membrane complexes for molecular dynamics simulations. PLoS One. 2:e880. 10.1371/journal.pone.0000880 17849009 PMC1963319

[bib37] Jo, S., T. Kim, V.G. Iyer, and W. Im. 2008. CHARMM-GUI: A web-based graphical user interface for CHARMM. J. Comput. Chem. 29:1859–1865. 10.1002/jcc.20945 18351591

[bib38] Jo, S., X. Cheng, S.M. Islam, L. Huang, H. Rui, A. Zhu, H.S. Lee, Y. Qi, W. Han, K. Vanommeslaeghe, . 2014. CHARMM-GUI PDB manipulator for advanced modeling and simulations of proteins containing nonstandard residues. Adv. Protein Chem. Struct. Biol. 96:235–265. 10.1016/bs.apcsb.2014.06.002 25443960 PMC4739825

[bib39] Jorgensen, W.L., J. Chandrasekhar, J.D. Madura, R.W. Impey, and M.L. Klein. 1983. Comparison of simple potential functions for simulating liquid water. J. Chem. Phys. 79:926–935. 10.1063/1.445869

[bib40] Ke, S., E.N. Timin, and A. Stary-Weinzinger. 2014. Different inward and outward conduction mechanisms in NaVMs suggested by molecular dynamics simulations. PLOS Comput. Biol. 10:e1003746. 10.1371/journal.pcbi.1003746 25079564 PMC4117422

[bib41] Kim, I., and T.W. Allen. 2011. On the selective ion binding hypothesis for potassium channels. Proc. Natl. Acad. Sci. USA. 108:17963–17968. 10.1073/pnas.1110735108 22011574 PMC3207655

[bib42] Kim, S., J. Lee, S. Jo, C.L. Brooks III, H.S. Lee, and W. Im. 2017. CHARMM-GUI ligand reader and modeler for CHARMM force field generation of small molecules. J. Comput. Chem. 38:1879–1886. 10.1002/jcc.24829 28497616 PMC5488718

[bib43] Klesse, G., S. Rao, M.S.P. Sansom, and S.J. Tucker. 2019. CHAP: A versatile tool for the structural and functional annotation of ion channel pores. J. Mol. Biol. 431:3353–3365. 10.1016/j.jmb.2019.06.003 31220459 PMC6699600

[bib44] Köpfer, D.A., C. Song, T. Gruene, G.M. Sheldrick, U. Zachariae, and B.L. de Groot. 2014. Ion permeation in K⁺ channels occurs by direct Coulomb knock-on. Science. 346:352–355. 10.1126/science.1254840 25324389

[bib45] Kohagen, M., M. Lepšík, and P. Jungwirth. 2014a. Calcium binding to calmodulin by molecular dynamics with effective polarization. J. Phys. Chem. Lett. 5:3964–3969. 10.1021/jz502099g 26276478

[bib46] Kohagen, M., P.E. Mason, and P. Jungwirth. 2014b. Accurate description of calcium solvation in concentrated aqueous solutions. J. Phys. Chem. B. 118:7902–7909. 10.1021/jp5005693 24802184

[bib47] Kopec, W., D.A. Köpfer, O.N. Vickery, A.S. Bondarenko, T.L.C. Jansen, B.L. de Groot, and U. Zachariae. 2018. Direct knock-on of desolvated ions governs strict ion selectivity in K^+^ channels. Nat. Chem. 10:813–820. 10.1038/s41557-018-0105-9 30030538

[bib48] Kwon, D.H., F. Zhang, Y. Suo, J. Bouvette, M.J. Borgnia, and S.Y. Lee. 2021. Heat-dependent opening of TRPV1 in the presence of capsaicin. Nat. Struct. Mol. Biol. 28:554–563. 10.1038/s41594-021-00616-3 34239123 PMC8335751

[bib49] Lee, J., X. Cheng, J.M. Swails, M.S. Yeom, P.K. Eastman, J.A. Lemkul, S. Wei, J. Buckner, J.C. Jeong, Y. Qi, . 2016. CHARMM-GUI input generator for NAMD, GROMACS, AMBER, OpenMM, and CHARMM/OpenMM simulations using the CHARMM36 additive force field. J. Chem. Theor. Comput. 12:405–413. 10.1021/acs.jctc.5b00935 PMC4712441 26631602

[bib50] Li, H., V. Ngo, M.C. Da Silva, D.R. Salahub, K. Callahan, B. Roux, and S.Y. Noskov. 2015. Representation of ion-protein interactions using the drude polarizable force-field. J. Phys. Chem. B. 119:9401–9416. 10.1021/jp510560k 25578354 PMC4516320

[bib51] Lindahl, E., M.J. Abraham, B. Hess, and D. van der Spoel. 2020. GROMACS 2020.2 manual.

[bib52] Liu, C., A. Zhang, N. Yan, and C. Song. 2021. Atomistic details of charge/space competition in the Ca^2+^ selectivity of ryanodine receptors. J. Phys. Chem. Lett. 12:4286–4291. 10.1021/acs.jpclett.1c00681 33909426

[bib53] Lomize, M.A., I.D. Pogozheva, H. Joo, H.I. Mosberg, and A.L. Lomize. 2012. OPM database and PPM web server: Resources for positioning of proteins in membranes. Nucleic Acids Res. 40:D370–D376. 10.1093/nar/gkr703 21890895 PMC3245162

[bib54] Marcus, Y. 1991. Thermodynamics of solvation of ions. Part 5—Gibbs free energy of hydration at 298.15 K. J. Chem. Soc. Faraday Trans. 87:2995–2999. 10.1039/FT9918702995

[bib55] McClendon, C.L., G. Friedland, D.L. Mobley, H. Amirkhani, and M.P. Jacobson. 2009. Quantifying correlations between allosteric sites in thermodynamic ensembles. J. Chem. Theor. Comput. 5:2486–2502. 10.1021/ct9001812 PMC2790287 20161451

[bib56] McGoldrick, L.L., A.K. Singh, K. Saotome, M.V. Yelshanskaya, E.C. Twomey, R.A. Grassucci, and A.I. Sobolevsky. 2018. Opening of the human epithelial calcium channel TRPV6. Nature. 553:233–237. 10.1038/nature25182 29258289 PMC5854407

[bib57] McKiernan, K.A., A.K. Koster, M. Maduke, and V.S. Pande. 2020. Dynamical model of the CLC-2 ion channel reveals conformational changes associated with selectivity-filter gating. PLOS Comput. Biol. 16:e1007530. 10.1371/journal.pcbi.1007530 32226009 PMC7145265

[bib58] Michaud-Agrawal, N., E.J. Denning, T.B. Woolf, and O. Beckstein. 2011. MDAnalysis: A toolkit for the analysis of molecular dynamics simulations. J. Comput. Chem. 32:2319–2327. 10.1002/jcc.21787 21500218 PMC3144279

[bib59] Millman, K.J., and M. Aivazis. 2011. Python for scientists and engineers. Comput. Sci. Eng. 13:9–12. 10.1109/MCSE.2011.36

[bib60] Morais-Cabral, J.H., Y. Zhou, and R. MacKinnon. 2001. Energetic optimization of ion conduction rate by the K^+^ selectivity filter. Nature. 414:37–42. 10.1038/35102000 11689935

[bib61] Moran, M.M. 2018. TRP channels as potential drug targets. Annu. Rev. Pharmacol. Toxicol. 58:309–330. 10.1146/annurev-pharmtox-010617-052832 28945977

[bib62] Nayal, M., and E. Di Cera. 1994. Predicting Ca^2+^-binding sites in proteins. Proc. Natl. Acad. Sci. USA. 91:817–821. 10.1073/pnas.91.2.817 8290605 PMC43040

[bib63] Neyton, J., and C. Miller. 1988. Discrete Ba^2+^ block as a probe of ion occupancy and pore structure in the high-conductance Ca^2+^ -activated K^+^ channel. J. Gen. Physiol. 92:569–586. 10.1085/jgp.92.5.569 3235974 PMC2228919

[bib64] Nilius, B., R. Vennekens, J. Prenen, J.G.J. Hoenderop, R.J.M. Bindels, and G. Droogmans. 2000. Whole-cell and single channel monovalent cation currents through the novel rabbit epithelial Ca^2+^ channel ECaC. J. Physiol. 527:239–248. 10.1111/j.1469-7793.2000.00239.x 10970426 PMC2270079

[bib65] Noskov, S.Y., S. Bernèche, and B. Roux. 2004. Control of ion selectivity in potassium channels by electrostatic and dynamic properties of carbonyl ligands. Nature. 431:830–834. 10.1038/nature02943 15483608

[bib66] Oliphant, T.E. 2007. Python for scientific computing. Comput. Sci. Eng. 9:10–20. 10.1109/MCSE.2007.58

[bib67] Owsianik, G., K. Talavera, T. Voets, and B. Nilius. 2006. Permeation and selectivity of TRP channels. Annu. Rev. Physiol. 68:685–717. 10.1146/annurev.physiol.68.040204.101406 16460288

[bib68] Palmer, C.P., X.-L. Zhou, J. Lin, S.H. Loukin, C. Kung, and Y. Saimi. 2001. A TRP homolog in *Saccharomyces cerevisiae* forms an intracellular Ca^2+^-permeable channel in the yeast vacuolar membrane. Proc. Natl. Acad. Sci. USA. 98:7801–7805. 10.1073/pnas.141036198 11427713 PMC35422

[bib69] Parrinello, M., and A. Rahman. 1981. Polymorphic transitions in single crystals: A new molecular dynamics method. J. Appl. Phys. 52:7182–7190. 10.1063/1.328693

[bib70] Patel, S. 2019. The secret life of calcium in cell signaling. Biochemist. 41:34–37. 10.1042/BIO04104034

[bib71] Pérez, F., and B.E. Granger. 2007. IPython: A system for interactive scientific computing. Comput. Sci. Eng. 9:21–29. 10.1109/MCSE.2007.53

[bib72] Peng, G., X. Shi, and T. Kadowaki. 2015. Evolution of TRP channels inferred by their classification in diverse animal species. Mol. Phylogenet. Evol. 84:145–157. 10.1016/j.ympev.2014.06.016 24981559

[bib73] Pethel, S.D., and D.W. Hahs. 2014. Exact test of independence using mutual information. Entropy. 16:2839–2849. 10.3390/e16052839

[bib74] Ramsey, I.S., M. Delling, and D.E. Clapham. 2006. An introduction to TRP channels. Annu. Rev. Physiol. 68:619–647. 10.1146/annurev.physiol.68.040204.100431 16460286

[bib75] Ringer, S. 1883. A further contribution regarding the influence of the different constituents of the blood on the contraction of the heart. J. Physiol. 4:29–42: 3. 10.1113/jphysiol.1883.sp000120 PMC1484842 16991336

[bib76] Sakipov, S., A.I. Sobolevsky, and M.G. Kurnikova. 2018. Ion permeation mechanism in epithelial calcium channel TRVP6. Sci. Rep. 8:5715. 10.1038/s41598-018-23972-5 29632318 PMC5890290

[bib77] Saotome, K., A.K. Singh, M.V. Yelshanskaya, and A.I. Sobolevsky. 2016. Crystal structure of the epithelial calcium channel TRPV6. Nature. 534:506–511. 10.1038/nature17975 27296226 PMC4919205

[bib78] Sauguet, L., F. Poitevin, S. Murail, C. Van Renterghem, G. Moraga-Cid, L. Malherbe, A.W. Thompson, P. Koehl, P.-J. Corringer, M. Baaden, and M. Delarue. 2013. Structural basis for ion permeation mechanism in pentameric ligand-gated ion channels. EMBO J. 32:728–741. 10.1038/emboj.2013.17 23403925 PMC3590989

[bib79] Schackert, F.K., J. Biedermann, S. Abdolvand, S. Minniberger, C. Song, A.J.R. Plested, P. Carloni, and H. Sun. 2022. Mechanism of calcium permeation in a glutamate receptor ion channel. ChemRxiv. 10.26434/chemrxiv-2022-x73hl-v2 PMC9976283 36758214

[bib80] Singh, A.K., L.L. McGoldrick, L. Demirkhanyan, M. Leslie, E. Zakharian, and A.I. Sobolevsky. 2019. Structural basis of temperature sensation by the TRP channel TRPV3. Nat. Struct. Mol. Biol. 26:994–998. 10.1038/s41594-019-0318-7 31636415 PMC6858569

[bib81] Stampe, P., and T. Begenisich. 1996. Unidirectional K^+^ fluxes through recombinant Shaker potassium channels expressed in single *Xenopus oocytes*. J. Gen. Physiol. 107:449–457. 10.1085/jgp.107.4.449 8722559 PMC2217009

[bib82] Thompson, A.N., I. Kim, T.D. Panosian, T.M. Iverson, T.W. Allen, and C.M. Nimigean. 2009. Mechanism of potassium-channel selectivity revealed by Na^+^ and Li^+^ binding sites within the KcsA pore. Nat. Struct. Mol. Biol. 16:1317–1324. 10.1038/nsmb.1703 19946269 PMC2825899

[bib83] Thomson, N.J., O.N. Vickery, C.M. Ives, and U. Zachariae. 2021. Ion-water coupling controls class A GPCR signal transduction pathways. bioRxiv. (Preprint posted August 28, 2020). 10.1101/2020.08.28.271510

[bib84] Tindjong, R., I. Kaufman, D.G. Luchinsky, P.V.E. McClintock, I.A. Khovanov, and R.S. Eisenberg. 2013. Stochastic dynamics of remote knock-on permeation in biological ion channels. 22nd Int. Conf. Noise Fluctuations:1–4. 10.1109/ICNF.2013.6578893

[bib85] Tominaga, M., M.J. Caterina, A.B. Malmberg, T.A. Rosen, H. Gilbert, K. Skinner, B.E. Raumann, A.I. Basbaum, and D. Julius. 1998. The cloned capsaicin receptor integrates multiple pain-producing stimuli. Neuron. 21:531–543. 10.1016/S0896-6273(00)80564-4 9768840

[bib86] Ulmschneider, M.B., C. Bagnéris, E.C. McCusker, P.G. Decaen, M. Delling, D.E. Clapham, J.P. Ulmschneider, and B.A. Wallace. 2013. Molecular dynamics of ion transport through the open conformation of a bacterial voltage-gated sodium channel. Proc. Natl. Acad. Sci. USA. 110:6364–6369. 10.1073/pnas.1214667110 23542377 PMC3631666

[bib87] Van Der Walt, S., S.C. Colbert, and G. Varoquaux. 2011. The NumPy array: A structure for efficient numerical computation. Comput. Sci. Eng. 13:22–30. 10.1109/MCSE.2011.37

[bib88] van Goor, M.K.C., J.G.J. Hoenderop, and J. van der Wijst. 2017. TRP channels in calcium homeostasis: From hormonal control to structure-function relationship of TRPV5 and TRPV6. Biochim. Biophys. Acta Mol. Cell Res. 1864:883–893. 10.1016/j.bbamcr.2016.11.027 27913205

[bib89] Van Goor, M.K., L. de Jager, Y. Cheng, and J. van der Wijst. 2020. High-resolution structures of TRPV channels: Unveiling a functionally diverse group of ion channels. Protein Sci. 29:1569–1580. 10.1002/pro.3861 32232875 PMC7314393

[bib90] Vanommeslaeghe, K., E. Hatcher, C. Acharya, S. Kundu, S. Zhong, J. Shim, E. Darian, O. Guvench, P. Lopes, I. Vorobyov, and A.D. Mackerell Jr. 2010. CHARMM general force field: A force field for drug-like molecules compatible with the CHARMM all-atom additive biological force fields. J. Comput. Chem. 31:671–690 19575467 10.1002/jcc.21367 PMC2888302

[bib91] Varma, S., D. Sabo, and S.B.K. Rempe. 2008. K^+^/Na^+^ selectivity in K channels and valinomycin: Over-coordination versus cavity-size constraints. J. Mol. Biol. 376:13–22. 10.1016/j.jmb.2007.11.059 18155244 PMC2390915

[bib92] Vennekens, R., J.G. Hoenderop, J. Prenen, M. Stuiver, P.H. Willems, G. Droogmans, B. Nilius, and R.J. Bindels. 2000. Permeation and gating properties of the novel epithelial Ca^2+^ channel. J. Biol. Chem. 275:3963–3969. 10.1074/jbc.275.6.3963 10660551

[bib93] Vögele, M., N.J. Thomson, S.T. Truong, and J. McAvity. 2021. PENSA 0.2.7 version v0.2.7. https://github.com/drorlab/pensa

[bib94] Vögele, M., N.J. Thomson, S.T. Truong, J. McAvity, U. Zachariae, and R.O. Dror. 2022. Systematic analysis of biomolecular conformational ensembles with PENSA. arXiv. 10.1063/5.0235544 PMC11698571 39745157

[bib95] Voets, T., A. Janssens, J. Prenen, G. Droogmans, and B. Nilius. 2003. Mg^2+^-dependent gating and strong inward rectification of the cation channel TRPV6. J. Gen. Physiol. 121:245–260. 10.1085/jgp.20028752 12601087 PMC2217333

[bib96] Waskom, M., O. Botvinnik, D. O’Kane, P. Hobson, J. Ostblom, S. Lukauskas, D.C. Gemperline, T. Augspurger, Y. Halchenko, J.B. Cole, . 2018. mwaskom/seaborn: v0.9.0 (July 2018).

[bib97] Watanabe, H., J. Vriens, A. Janssens, R. Wondergem, G. Droogmans, and B. Nilius. 2003. Modulation of TRPV4 gating by intra- and extracellular Ca^2+^. Cell Calcium. 33:489–495. 10.1016/S0143-4160(03)00064-2 12765694

[bib98] Wu, E.L., X. Cheng, S. Jo, H. Rui, K.C. Song, E.M. Davila-Contreras, Y. Qi, J. Lee, V. Monje-Galvan, R.M. Venable, . 2014. CHARMM-GUI membrane builder toward realistic biological membrane simulations. J. Comput. Chem. 35:1997–2004. 10.1002/jcc.23702 25130509 PMC4165794

[bib99] Yue, L., J.B. Peng, M.A. Hediger, and D.E. Clapham. 2001. CaT1 manifests the pore properties of the calcium-release-activated calcium channel. Nature. 410:705–709. 10.1038/35070596 11287959

[bib100] Zhang, F., S.M. Hanson, A. Jara-Oseguera, D. Krepkiy, C. Bae, L.V. Pearce, P.M. Blumberg, S. Newstead, and K.J. Swartz. 2016. Engineering vanilloid-sensitivity into the rat TRPV2 channel. eLife. 5. e16409. 10.7554/eLife.16409 27177419 PMC4907692

[bib101] Zhang, A., H. Yu, C. Liu, and C. Song. 2020. The Ca^2+^ permeation mechanism of the ryanodine receptor revealed by a multi-site ion model. Nat. Commun. 11:922. 10.1038/s41467-020-14573-w 32066742 PMC7026163

[bib102] Zhou, X.-L., A.F. Batiza, S.H. Loukin, C.P. Palmer, C. Kung, and Y. Saimi. 2003. The transient receptor potential channel on the yeast vacuole is mechanosensitive. Proc. Natl. Acad. Sci. USA. 100:7105–7110. 10.1073/pnas.1230540100 12771382 PMC165837

