# Supplementary material for: A cooperative knock-on mechanism underpins Ca2+-selective cation permeation in TRPV channels
Source: J Gen Physiol. 2023 Mar 21;155(5):e202213226. doi: 10.1085/jgp.202213226 (PMC10038842; doi:10.1085/jgp.202213226)
Supplement: Table S1 — shows summary of simulation details of Ca2+-selective TRPV channels. [file JGP_202213226_TableS1.docx]

Table S1: Summary of simulation details of Ca^2+^-selective TRPV channels.

| **Protein** | TRPV5 | | | TRPV6 | | |
| --- | --- | --- | --- | --- | --- | --- |
| **Structure** | 6DMU  (262-639 & PI(4,5)P_2_) | | | 6BO8  (262-638) | | |
| **Force field** | CHARMM36m | | | CHARMM36m | | |
| **Water** | TIP3P | | | TIP3P | | |
| **Ligand** | PI(4,5)P_2_ (CGenFF) | | | - | | |
| **Ion** | **150 mM CaCl_2_**  291 Ca^2+^ (Zhang *et al.*) 574 Cl^-^ (CHARMM36m) | **150 mM NaCl**  295 Na^+^ (CHARMM36m)  287 Cl^-^ (CHARMM36m) | **75 mM CaCl_2_ + 75mm NaCl**  143 Ca^2+^ (Zhang *et al.*) 151 Na^+^ (CHARMM36m)  429 Cl^-^ (CHARMM36m) | **150 mM CaCl_2_**  279 Ca^2+^ (Zhang *et al.*) 562 Cl^-^ (CHARMM36m) | **150 mM NaCl**  279 Na^+^ (CHARMM36m)  283 Cl^-^ (CHARMM36m) | **75 mM CaCl_2_ + 75mm NaCl**  140 Ca^2+^ (Zhang *et al.*) 140 Na^+^ (CHARMM36m)  424 Cl^-^ (CHARMM36m) |
| **Independent simulations** | 5 | 5 | 5 | 5 | 5 | 5 |
| **Total simulation time (***µ***s)** | 1.25 | 1.25 | 1.25 | 1.25 | 1.25 | 1.25 |
| **Estimated voltage (mV)** | -410 | -410 | -410 | -410 | -410 | -410 |
| **Permeation events** | 85 | 165 | 165 | 189 | 197 | 49 |
