# Supplementary material for: A cooperative knock-on mechanism underpins Ca2+-selective cation permeation in TRPV channels
Source: J Gen Physiol. 2023 Mar 21;155(5):e202213226. doi: 10.1085/jgp.202213226 (PMC10038842; doi:10.1085/jgp.202213226)
Supplement: Table S2 — shows summary of simulation details of non-selective TRPV channels. [file JGP_202213226_TableS2.docx]

Table S2: Summary of simulation details of non-selective TRPV channels.

| **Protein** | TRPV2 | | | TRPV3 | | |
| --- | --- | --- | --- | --- | --- | --- |
| **Structure** | 6BO4  (327-691) | | | 6PVP  (375-745) | | |
| **Force field** | CHARMM36m | | | CHARMM36m | | |
| **Water** | TIP3P | | | TIP3P | | |
| **Ligand** | - | | | - | | |
| **Ion** | **150 mM CaCl_2_**  253 Ca^2+^ (Zhang *et al.*) 526 Cl^-^ (CHARMM36m) | **150 mM NaCl**  253 Na^+^ (CHARMM36m)  273 Cl^-^ (CHARMM36m) | **75 mM CaCl_2_ + 75mm NaCl**  127 Ca^2+^ (Zhang *et al.*) 127 Na^+^ (CHARMM36m)  401 Cl^-^ (CHARMM36m) | **150 mM CaCl_2_**  291 Ca^2+^ (Zhang *et al.*) 582 Cl^-^ (CHARMM36m) | **150 mM NaCl**  291 Na^+^ (CHARMM36m)  291 Cl^-^ (CHARMM36m) | **75 mM CaCl_2_ + 75mm NaCl**  146 Ca^2+^ (Zhang *et al.*) 146 Na^+^ (CHARMM36m)  438 Cl^-^ (CHARMM36m) |
| **Independent simulations** | 5 | 5 | 5 | 5 | 5 | 5 |
| **Total simulation time (***µ***s)** | 1.25 | 1.25 | 1.25 | 1.25 | 1.25 | 1.25 |
| **Estimated voltage (mV)** | -410 | -410 | -410 | -410 | -410 | -410 |
| **Permeation events** | 60 | 59 | 72 | 941 | 309 | 441 |
