# Supplementary material for: A cooperative knock-on mechanism underpins Ca2+-selective cation permeation in TRPV channels
Source: J Gen Physiol. 2023 Mar 21;155(5):e202213226. doi: 10.1085/jgp.202213226 (PMC10038842; doi:10.1085/jgp.202213226)
Supplement: Table S3 — shows summary of simulation details of additional control simulations of Ca2+-selective TRPV channels. [file JGP_202213226_TableS3.docx]

Table S3: Summary of simulation details of additional control simulations of Ca^2+^-selective TRPV channels.

| **Protein** | TRPV5 | | | |
| --- | --- | --- | --- | --- |
| **Structure** | 6DMU  (262-639  PI(4,5)P2) | | | |
| **Force field** | CHARMM36m | | | |
| **Water** | TIP3P | | | |
| **Ligand** | PI(4,5)P2 (CGenFF) | | | PI(4,5)P2 deprotonated (CGenFF) |
| **Ion** | 75 mM CaCl_2_ + 75mm NaCl  143 Ca^2+^ (Zhang *et al*.) 151 Na^+^ (CHARMM36m)  429 Cl^-^ (CHARMM36m) | 25 mM CaCl_2_  52 Ca^2+^ (Zhang *et al*.) 96 Cl^-^ (CHARMM36m) | 150 mM CaCl_2_  291 Ca^2+^ (CHARMM36m)  574 Cl^-^ (CHARMM36m) | 150 mM NaCl  311 Na^+^ (CHARMM36m)  287 Cl^-^ (CHARMM36m) |
| **Independent simulations** | 3 | 3 | 3 | 3 |
| **Total simulation time (***µ***s)** | 0.75 | 0.3 | 0.75 | 0.45 |
| **Estimated voltage (mV)** | -205 | -410 | -410 | -410 |
| **Total permeations** | 22 | 2 | 0 | 95 |
