# Supplementary material for: A cooperative knock-on mechanism underpins Ca2+-selective cation permeation in TRPV channels
Source: J Gen Physiol. 2023 Mar 21;155(5):e202213226. doi: 10.1085/jgp.202213226 (PMC10038842; doi:10.1085/jgp.202213226)
Supplement: Table S4 — shows average time to permeate through the TRPV pore, as defined by the z position between binding sites A and C. [file JGP_202213226_TableS4.docx]

Table S4: Average time to permeate through the TRPV pore, as defined by the *z* position between binding sites A and C. The mean permeation time and standard error of the mean were calculated from five-fold replicated 250 ns simulations in mono-cationic 150 mM CaCl_2_ or 150 mM NaCl.

|  | **Permeation time (ns)** | |
| --- | --- | --- |
|  | **Ca2+** | **Na+** |
| **TRPV2** | 28.4 *±* 7.6 | 7.0 *±* 1.8 |
| **TRPV3** | 6.3 *±* 1.2 | 2.8 *±* 0.2 |
| **TRPV5** | 28.4 *±* 3.9 | 18.2 *±* 3.8 |
| **TRPV6** | 12.0 *±* 1.0 | 12.1 *±* 1.5 |
