# Supplementary material for: A cooperative knock-on mechanism underpins Ca2+-selective cation permeation in TRPV channels
Source: J Gen Physiol. 2023 Mar 21;155(5):e202213226. doi: 10.1085/jgp.202213226 (PMC10038842; doi:10.1085/jgp.202213226)
Supplement: Table S5 — shows calculated exSSI and exSSInorm values of cation transition from binding sites in MD simulations of TRPV channels. [file JGP_202213226_TableS5.docx]

Table S5: Calculated *exSSI* and *exSSI_norm_* values of cation transition from binding sites in MD simulations of TRPV channels. The mean *exSSI* or *exSSI_norm_*, and standard error of the mean, were calculated from five-fold replicated 250 ns simulations in mono-cationic solutions of 150 mM CaCl_2_ or 150 mM NaCl.

|  | **Cation** | *exSSI*(*A, B*) | *exSSI*(*A, B*)*_norm_* | *exSSI*(*B,C*) | *exSSI*(*B,C*)*_norm_* |
| --- | --- | --- | --- | --- | --- |
| **TRPV2** | Ca2+ | 0.15 *±* 0.06 | 0.12 *±* 0.05 | 0.88 *±* 0.13 | 0.44 *±* 0.03 |
|  | Na^+^ | 0.26 *±* 0.13 | 0.18 *±* 0.06 | 0.13 *±* 0.03 | 0.18 *±* 0.02 |
| **TRPV3** | Ca2+ | 0.34 *±* 0.09 | 0.17 *±* 0.04 | 0.96 *±* 0.17 | 0.32 *±* 0.05 |
|  | Na^+^ | 0.05 *±* 0.04 | 0.03 *±* 0.02 | 0.26 *±* 0.16 | 0.10 *±* 0.06 |
| **TRPV5** | Ca2+ | 1.25 *±* 0.15 | 0.50 *±* 0.05 | 1.01 *±* 0.25 | 0.40 *±* 0.06 |
|  | Na^+^ | 1.05 *±* 0.31 | 0.30 *±* 0.06 | 0.84 *±* 0.17 | 0.28 *±* 0.04 |
| **TRPV6** | Ca2+ | 0.81 *±* 0.16 | 0.31 *±* 0.03 | 0.53 *±* 0.25 | 0.27 *±* 0.04 |
|  | Na^+^ | 1.55 *±* 0.18 | 0.39 *±* 0.03 | 0.88 *±* 0.10 | 0.32 *±* 0.03 |
