# Supplementary material for: A cooperative knock-on mechanism underpins Ca2+-selective cation permeation in TRPV channels
Source: J Gen Physiol. 2023 Mar 21;155(5):e202213226. doi: 10.1085/jgp.202213226 (PMC10038842; doi:10.1085/jgp.202213226)
Supplement: Table S6 — shows selectivity ratios of Ca2+ and Na+ permeation events from simulations of TRPV channels in a dicationic solution. [file JGP_202213226_TableS6.docx]

Table S6: Selectivity ratios of Ca^2+^ and Na^+^ permeation events from simulations of TRPV channels in a di-cationic solution.

|  | *PCa/PNa* |
| --- | --- |
| **TRPV2** | 5.0 |
| **TRPV3** | 1.7 |
| **TRPV5** | 2.6 |
| **TRPV6** | 4.4 |
