# Supplementary material for: A cooperative knock-on mechanism underpins Ca2+-selective cation permeation in TRPV channels
Source: J Gen Physiol. 2023 Mar 21;155(5):e202213226. doi: 10.1085/jgp.202213226 (PMC10038842; doi:10.1085/jgp.202213226)
Supplement: Table S7 — shows RMSF of the backbone of SF residues of TRPV channels from MD simulations. [file JGP_202213226_TableS7.docx]

Table S7: Root mean square fluctuation (RMSF) of the backbone of SF residues of TRPV channels from MD simulations. The mean RMSF and standard error of the mean for each residue was calculated from five-fold replicated 250 ns simulations of each channel in 150 mM CaCl_2_.

|  | **Selectivity filter residue RMSF (A˚ )** | | | |
| --- | --- | --- | --- | --- |
|  | **alpha** | **beta** | **gamma** | **delta** |
| **TRPV2** | 0.8 *±* 0.04 | 0.7 *±* 0.04 | 0.6 *±* 0.05 | 0.7 *±* 0.05 |
| **TRPV3** | 0.9 *±* 0.04 | 0.8 *±* 0.05 | 0.7 *±* 0.02 | 0.7 *±* 0.02 |
| **TRPV5** | 1.0 *±* 0.07 | 0.7 *±* 0.04 | 0.7 *±* 0.03 | 0.7 *±* 0.02 |
| **TRPV6** | 1.3 *±* 0.08 | 0.9 *±* 0.04 | 0.9 *±* 0.04 | 0.9 *±* 0.03 |
